# The Tudor Domain-Containing Protein, Kotsubu (CG9925), Localizes to the Nuage and Functions in piRNA Biogenesis in *D. melanogaster*

**DOI:** 10.3389/fmolb.2022.818302

**Published:** 2022-03-29

**Authors:** Lin-Xenia Lim, Wakana Isshiki, Taichiro Iki, Shinichi Kawaguchi, Toshie Kai

**Affiliations:** Graduate School of Frontier Biosciences, Osaka University, Suita, Japan

**Keywords:** Tudor domain, nuage, piNG-body, piRNA, CG9925, stellate

## Abstract

Silencing of transposable elements (TEs) by Piwi-interacting RNAs (piRNAs) is crucial for maintaining germline genome integrity and fertility in animals. To repress TEs, PIWI clade Argonaute proteins cooperate with several Tudor domain-containing (Tdrd) proteins at membraneless perinuclear organelles, called nuage, to produce piRNAs to repress transposons. Here, we identify and characterize Kotsubu (Kots), one of the *Drosophila* Tudor domain-containing protein-1 (Tdrd1) orthologs, encoded by the *CG9925* gene, that localizes to the nuage in gonads. We further show the dynamic localization of Kots in the male germline, where it shows perinuclear signals in spermatogonia but forms large cytoplasmic condensates in the spermatocytes that overlap with components of piNG-body, a nuage-associated organelle. The loss of *kots* results in a notable upregulation of *stellate* and a corresponding reduction in the *suppressor of stellate* piRNAs in the mutants. Furthermore, a moderate yet significant reduction of other piRNAs was observed in *kots* mutant testes. Taken together, we propose that Kots functions in the piRNA pathway, predominantly in the male germline by forming discrete cytoplasmic granules.

## Introduction

Transposons, or transposable elements (TEs), are repetitive genetic sequences that occupy large proportions in animal genomes—at least 20% in flies, 40% in mice, and 45% in humans (Huang et al., 2012; Wang and Kunze, 2015; McCullers and Steiniger, 2017). With the ability to drive its own expression and relocate within the host genome, TEs can induce genomic aberrations such as insertions, deletions, and duplications, which can compromise genetic stability and lead to disorders and genetic diseases (Payer and Burns, 2019). Hence, TE activities must be tightly regulated by the host organism.

In gonadal cells, the control of TEs largely relies on silencing pathways mediated by a class of small non-coding RNAs, namely, the PIWI-interacting RNAs (piRNAs) (Aravin et al., 2006; Girard et al., 2006; Grivna et al., 2006; Lau et al., 2006; Vagin et al., 2006). These 23- to 29-nucleotide (nt) RNAs bind to the PIWI clade Argonaute proteins, Piwi, Aubergine (Aub), and Argonaute-3 (Ago3) in *Drosophila*, forming piRNA-induced silencing complexes (piRISCs) and to direct the transcriptional and posttranscriptional silencing of TEs (Parker et al., 2004; Aravin et al., 2006; Girard et al., 2006; Grivna et al., 2006; Gunawardane et al., 2007; Darricarrére et al., 2013). Piwi, Aub, and Ago3 are spatially and functionally distinct in their roles in silencing transposons. The binding of piRNAs translocates Piwi into the nucleus, where it exerts co-transcriptional silencing of TE sequences by recruiting factors required for heterochromatin silencing and biogenesis of primary piRNAs (Klenov et al., 2014; Iwasaki et al., 2016; Fabry et al., 2019; Schnabl et al., 2021). In the cytoplasm of germline cells, amplification of piRNAs is a germline-exclusive mechanism which involves piRNA-directed cleavage of TE transcripts by Aub and Ago3 (Brennecke et al., 2007; Gunawardane et al., 2007). This amplification, known as the ping-pong cycle, is thought to take place at membraneless perinuclear structures called nuage (Eddy, 1976; Saffman and Lasko, 1999; Lim and Kai, 2007).

Robust piRNA biogenesis and defense against transposons are coordinated by a repertoire of proteins, including those from the aforementioned PIWIs and Tudor domain-containing family proteins (Tdrds). Tdrd family members are characterized by either a single or multiple Tudor domains, each consists of approximately 60 amino acids that fold into three to five antiparallel β-sheets to form a barrel-like core structure (Selenko et al., 2001; Lasko, 2010). Many Tudor domains of Tdrds expressed in the gonads have additional α-helices and β-sheets, giving rise to an extended Tudor (eTud) domain of approximately 180 amino acids (Friberg et al., 2009; Liu et al., 2010a; Chen et al., 2011). Tudor domains selectively recognize symmetrically dimethylated arginine (sDMA) or lysine residues, where methylarginine interactions are considered predominant in RNA metabolism, including piRNA pathways (Nishida et al., 2009; Vagin et al., 2009; Liu et al., 2010b; Kirino et al., 2010; Ku and Lin, 2014). As demonstrated in a recent study in human cell culture systems, these DMA–Tudor interactions underlie the complex control of condensate formation in cells (Courchaine et al., 2021), implying that Tdrds involved in piRNA production may play a role in nuage formation.

Mouse testes and *Drosophila* ovaries are excellent models for studying piRNA pathways in vertebrates and invertebrates, respectively. Studies using these systems have shown that perturbations in piRNA biogenesis result in substantial derepression of TEs in the germlines, often leading to infertility in animals. Several Tdrd proteins that function in the piRNA pathway have been identified and characterized in these systems. However, details of the molecular mechanism of piRNA-mediated TE silencing in the *Drosophila* testicular system remains poorly understood (Quénerch’du et al., 2016; Chen et al., 2021).

**FIGURE 1:**
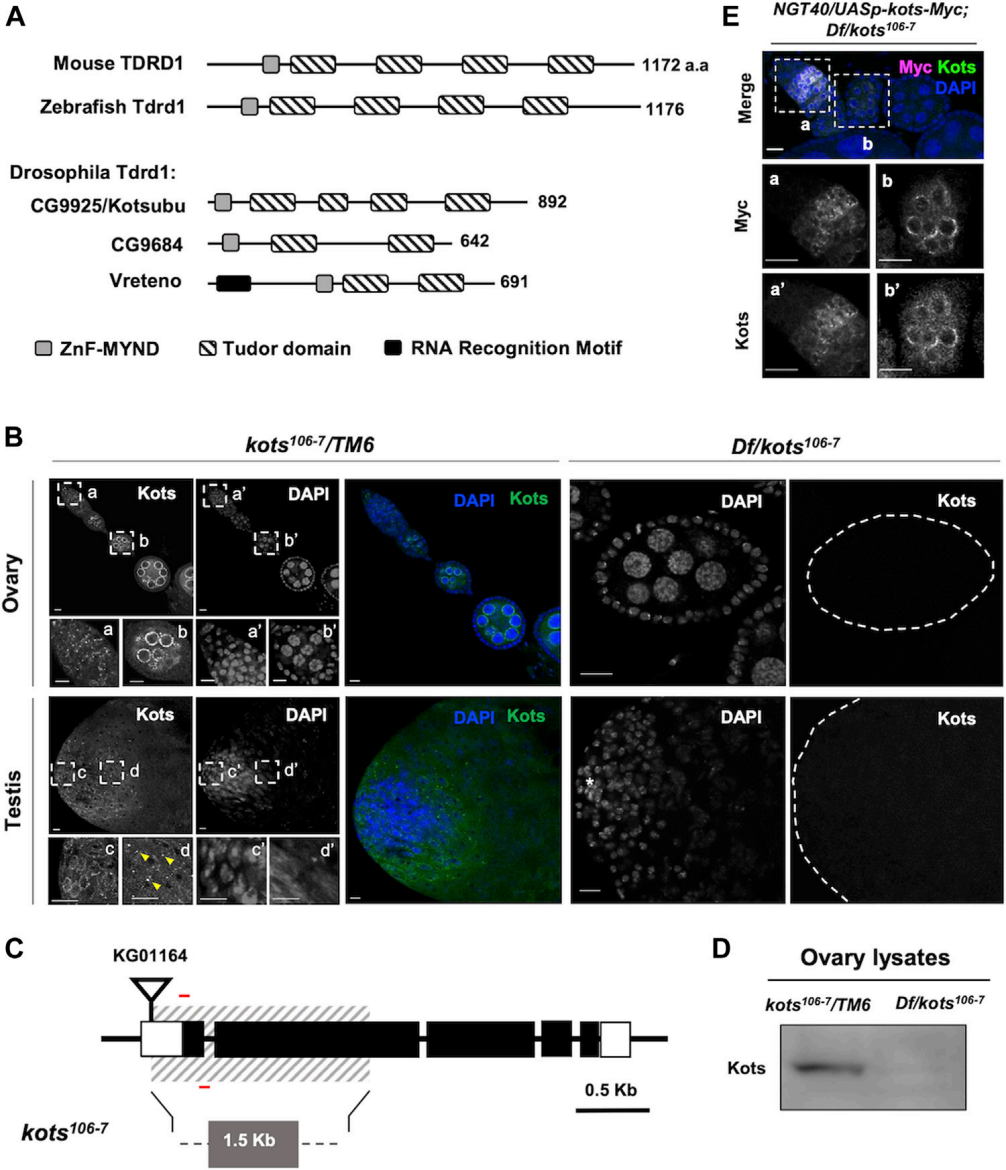
*Drosophila* CG9925/Kotsubu localizes to the nuclear periphery in germline cells. **(A)** Structural diagram depicting protein domains of Tdrd1 orthologs in mice, zebra fish, and flies. MYND-type zinc finger domain and Tudor domains are in light gray and stripes, respectively. In fly, an additional N-terminal RNA recognition motif (black) is found in Vreteno but is absent in the other orthologous proteins. **(B)** Kots is observed as perinuclear foci in germline cells in the germaria (a) and localizes to the nuclear peripheral nuage in the egg chambers (b) in ovaries. In testes, Kots appears as perinuclear foci in the spermatogonia (c) and condenses into enlarged foci (yellow arrowheads) in the spermatocytes (d). Endogenous Kots is absent in deficiency (Df) over *kots* mutant gonads *(Df/kots* ^*106-7*^ *)*. **(C)** Loss-of-function allele of *kots* was generated by imprecise excision of a P-element insertion, *KG01164*. Red bars represent the primers used to detect 118 bp of the first exon. **(D)** Western blotting of ovarian lysates showing a single band for Kots in the heterozygous control that is absent in Df/*kots* ^*106-7*^ mutants. **(E)** Immunostaining of *kots* mutant ovaries expressing C-terminal Myc-tagged Kots (Kots-Myc) in germline cells with anti-Myc and Kots antibodies. *NGT40* was used to drive the expression of UASp*-kots-Myc* in the germline. Perinuclear puncta in the germaria (a, a’) and egg chambers (b, b’) are discernible. All scale bars are 10 μm.

In this study, we identify and characterize *CG9925*, the ortholog of *Tdrd1* in humans, mice, and zebra fish, as a piRNA component in *Drosophila*. In vertebrate models, Tdrd1 possesses an N-terminal MYND zinc finger, followed by four tandem Tudor domains (Figure 1A) (Chuma et al., 2003; Chuma et al., 2006). Earlier studies in mice demonstrated the interaction between Tdrd1 and Mili, a mouse PIWI protein, which is required for TE suppression and male fertility (Chuma et al., 2003; Chuma et al., 2006). Unlike its vertebrate counterparts, the *Drosophila* genome contains three *Tdrd1* orthologs, *CG9684*, *CG9925*, and *Vreteno (Vret)*, raising the question whether each of these orthologs plays a differential role in the piRNA pathway. Of the three orthologs, Vret has been shown to be required for primary piRNA biogenesis in both germline and somatic cells in the ovary, while CG9684 is less understood, although it has a minor role in female fertility (Handler et al., 2011).

Here, we show that unlike Vret, endogenous CG9925 is expressed exclusively in the germline and localizes at the perinuclear nuage in ovaries and testes. In addition to the nuage, CG9925 exhibits distinct localization that is regulated in a temporal manner at different stages of spermatogenesis. The loss-of-function allele of *CG9925* displayed more severe defects in piRNA biogenesis in testes than in ovaries. Taken together, we propose that the *Drosophila* Tdrd1 ortholog CG9925 is differentially regulated in the ovaries and testes, where it exerts a male-dominant role in piRNA biogenesis.

## Results

### A Tudor Domain-Containing Protein Encoded by CG9925, Kotsubu, Localizes to the Nuage in Germline Cells

*Drosophila CG9925* encodes a protein comprising an MYND-type zinc finger motif and four extended Tudor domains (Handler et al., 2011), showing the closest structural similarity to Tdrd1 in mice and zebra fish among the three Tdrd1 orthologs in *Drosophila* (Figure 1A). Immunostaining with polyclonal antibody generated against the C terminus of CG9925 revealed its expression in germline cells of ovaries and testes, where its perinuclear localization resembles the nuage (Figure 1B) (Eddy, 1976; Lim and Kai, 2007). In support of this, CG9925 was found to co-localize with Ago3, a PIWI family protein that functions in piRNA biogenesis at the nuage (Supplementary Figure 1A). Notably, in testes, CG9925 not only localizes to the nuage but also exhibits a distinct localization pattern. During spermatogenesis, germline stem cells (GSCs) divide asymmetrically to give rise to a daughter GSC and a differentiating gonialblast (GB) (Fuller, 1993). GBs undergo four mitotic divisions to produce spermatogonia, each of which differentiates into primary spermatocytes and eventually haploid spermatids through meiosis. In spermatogonia, CG9925 co-localizes with nuage components at the nuclear periphery (Figure 1B inset c). However, during the transition from spermatogonia to spermatocytes, the nuage-localized signal was largely reduced. Instead, aggregates containing CG9925 were observed on the cytoplasmic side of the nucleus (Figure 1B inset d). Because of this unique grain-like structure, we named *CG9925* “*kotsubu* (*kots*),” the Japanese meaning of “small grain.”

To further address the dynamics of Kots subcellular localization during spermatogenesis, we stained Kots in the loss-of-function alleles of *bag of marbles* (*bam* ^*∆86*^) and *cannonball* (*can* ^*12*^), which arrest germline cell differentiation at the spermatogonia and spermatocyte stages, respectively (Ohlstein et al., 2000). In *bam* ^*∆86*^ testes accumulating spermatogonial cells, Kots was found at the nuage with minimal focal aggregation (Supplementary Figure 1B). In contrast, large aggregates of Kots persisted in the distal parts of *can* ^*12*^ testes containing an expanded region of primary spermatocytes (Supplementary Figure 1B). These results indicate that punctate aggregates of Kots are a feature of primary spermatocytes. This stage-dependent localization of Kots in the testes was not observed in the ovaries (Supplementary Figure 1A), indicating a male-specific spatiotemporal regulation of Kots during spermatogenesis.

Kots aggregates in spermatocytes resemble previously reported cytoplasmic germ granules termed the piRNA nuage giant (piNG) bodies, structures enriched with piRNA factors such as Vasa, Aub, and Ago3 in primary spermatocytes (Kibanov et al., 2011). Co-immunostaining with Ago3 and Aub showed overlapping signals in the cytoplasm (Supplementary Figure 1C), suggesting that Kots is a component of piNG-bodies.

### Loss of Kots Results in the Derepression of *Stellate* in the Male Germline

To further characterize the function of Kots in the germline, we generated a null allele (*kots* ^*106-7*^) by imprecise excision of a P-element inserted at the 5’ UTR (Figure 1C). An approximate 1.57-kb deletion, which spans the first and most of the second exons, resulted in a loss-of-function allele. The loss of functional transcripts in both female and male germlines was verified using quantitative PCR (qPCR) (Supplementary Figure 2A). Consistently, in the mutant ovaries and testes (*Df(3R)Exel6171/kots* ^*106-7*^), endogenous Kots was undetectable (Figure 1B). Additionally, Western blot analysis of ovarian lysate showed a single band corresponding to the expected size of Kots in the heterozygous control, which was absent in the mutant ovaries, hence verifying the loss-of-function allele (Figure 1D, Supplementary Figure 1D). Subsequently, we generated a transgene expressing *kots-myc* placed under the *UAS* and expressed using the germline driver *NGT40* (Tracey et al., 2000). Immunostaining of ovaries expressing Myc-tagged Kots with anti-Myc antibody showed perinuclear signals in ovarian germline cells, further supporting that Kots localizes to the nuage (Figure 1E).

**FIGURE 2:**
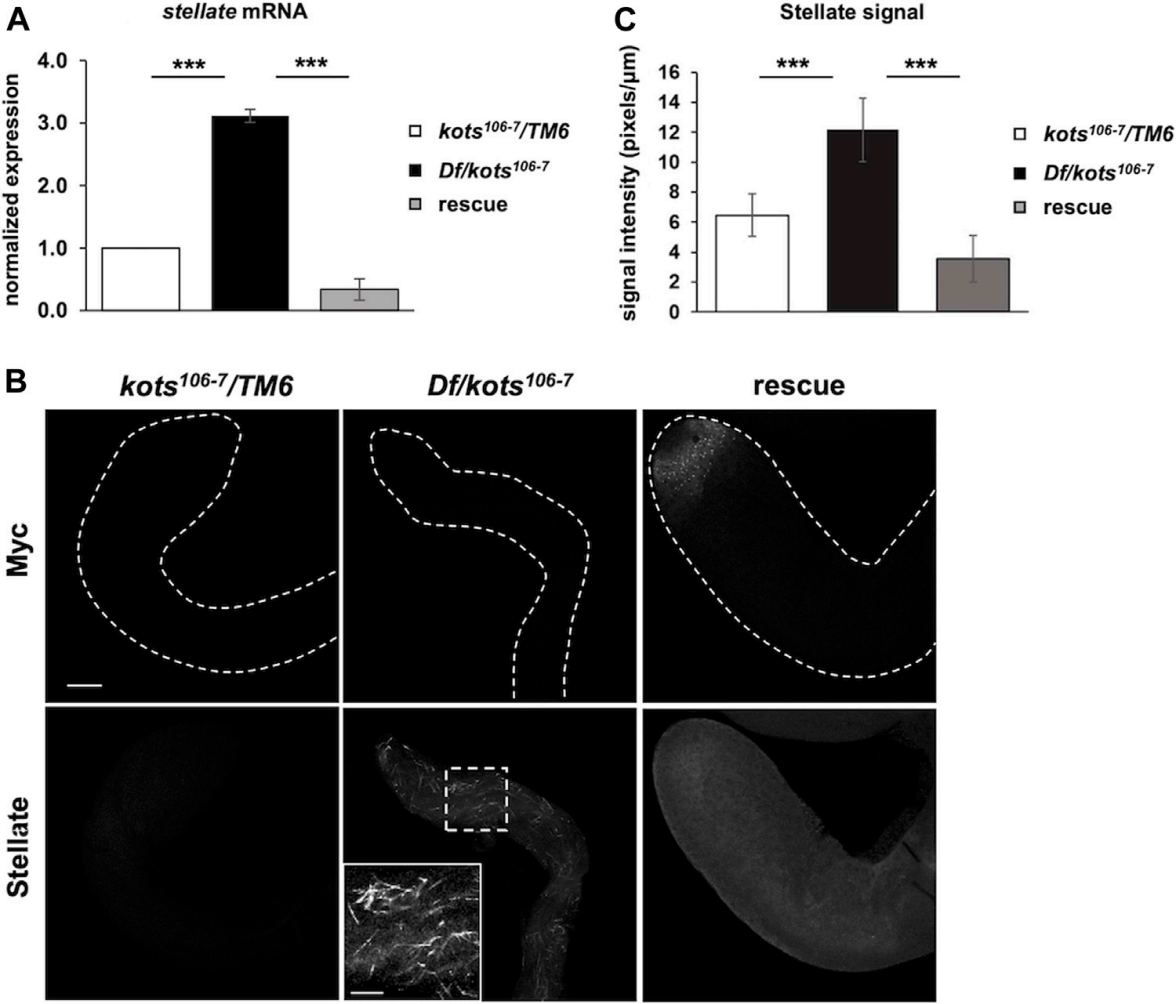
Loss of *kots* results in upregulation of *stellate* in testes. **(A)** Derepression of *stellate (ste)* in loss-of-function *kots* testes and re-repression in flies expressing Kots-Myc in *kots* mutant background (*n* = 3, *t*-test, *p*-value < 0.005, error bars indicate s.d.). *kots* heterozygous testes were used as the control. **(B)** Robust upregulation of *stellate (Ste)* protein in *kots* mutant testes and re-repression upon expression of UASp*-kots-Myc* in testicular germline cells by the *bam-Gal4* driver. Inset shows a magnified image of *Ste* protein in mutant testes. *kots* heterozygous testes were used as the control. All scale bars are 20 μm. **(C)** Quantification of *Ste* signal intensity in loss-of-function *kots* testes. Signal intensities were measured for independent regions (*n* = 3) from each sample. The average signal intensities of *Ste* were normalized against the Myc signal. Increment of the *Ste* signal was observed in mutant testes compared to the control and was re-repressed in the rescue (*t*-test, *p*-value < 0.05, error bars indicate s.d.).

Despite the loss of function, *kots* transheterozygous over deficiency mutants were viable, and the morphology of ovaries and testes was comparable to that of the control (Supplementary Figure 2B). No significant difference was observed in the egg hatching rate or total sperm count between *kots* mutants and the controls, suggesting a minor role of Kots in development and fertility (Supplementary Figures 2C, D). Nevertheless, the localization of Kots prompted us to further explore its function in the piRNA pathway. In *Drosophila* testes, it is known that *stellate* (*ste*), encoded on the X-chromosome (FlyBase ID FBgn0003523), is normally repressed by piRNAs derived from *suppressor of stellate (Su(Ste))* repeats on the Y-chromosome (Balakireva et al., 1992; Palumbo et al., 1994; Bozzetti et al., 2012). Indeed, Kots mutants showed 3-fold upregulation of *ste* mRNA (Figure 2A). Correspondingly, we observed the robust accumulation of *ste* protein in the spermatocytes of mutant testes, which strongly suggests that *kots* functions in silencing *ste* through the piRNA pathway (Figure 2B). In support of this, quantitative analysis of *ste* signal showed a significant difference in the signal intensity between the control and loss-of-function *kots* testes (Figure 2C). Derepression of *ste* was restored to that in the control level upon overexpression of Myc-tagged Kots driven by *bam-Gal4* (Chen and McKearin, 2003) (Figure 2A), along with the loss of *ste* crystals in late spermatogonia and spermatocytes, and a reduction in *ste* signal intensity (Figure 2C), suggesting the role of *kots* in the suppression of *ste*.

### Kots Interacts, Genetically and Physically, With Other piRNA Components

**FIGURE 3:**
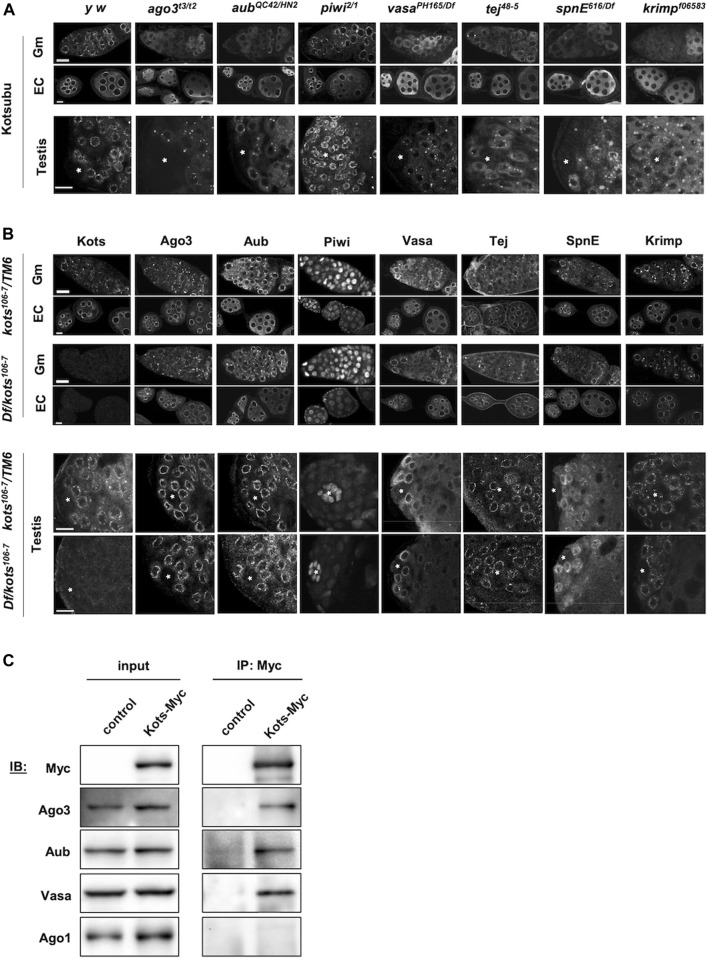
*kots* interacts genetically and physically with components of the piRNA pathway. **(A)** Immunostaining of Kots in piRNA pathway mutant ovaries (top panel: germaria, Gm; egg chambers, EC) and testes (bottom panel). Asterisks indicate the testicular hub at the apical region. All scale bars are 10 μm. **(B)** Immunostaining of piRNA pathway components in *kots* mutant ovaries and testes. All scale bars are 10 μm. **(C)** Immunoprecipitation of Kots-Myc expressed in germline cells in ovaries. *NGT40*; *nos-Gal4-VP16* was used to drive the expression of UASp-*kots-Myc*. Western blots (IB) detecting piRNA pathway components showing co-precipitation of two PIWI family proteins involved in the ping-pong cycle and Vasa but not miRNA pathway factor, Ago1. *y w* ovaries devoid of UASp-*kots-Myc* transgene were used as the control.

piRNA pathway components are known to interact and assemble in a hierarchical order, for example, Vasa localizes at the nuage and is required for downstream assembly of piRNA pathway components such as Aub and Ago3 (Lim and Kai, 2007; Nagao et al., 2011). To understand the interactions of Kots with other components in the pathway, we examined the localization of Kots in different piRNA pathway mutants. In *piwi* mutants where the primary piRNA processing, but not ping-pong amplification, were perturbed, Kots exhibited perinuclear nuage localization in the germaria and egg chambers like that in the controls (Figure 3A). However, dispersed cytoplasmic signal of Kots was observed in the mutant germaria of *ago3* and *aub*, whose proteins localize at the nuage and function in ping-pong amplification. This defect was also observed in the absence of other nuage components such as the DEAD-box helicase *vasa (vas)*, Tudor domain proteins *spindle-E* (*spnE*), *tejas (tej)*, and *krimper (krimp)* which are known to be important factors for piRNA biogenesis *via* the ping-pong cycle (Lim and Kai, 2007; Patil and Kai, 2010). In these ping-pong cycle mutants, except *piwi*, perinuclear Kots in later stages of egg chambers was also displaced from the nuage, albeit to a lesser extent in *aub* mutants. In a similar manner, Kots localization was affected in the early stages of spermatogenesis. With the exception of *piwi* Kots was highly dispersed from the nuage in spermatogonial cells and formed large aggregates in spermatocytes of piRNA pathway mutants (Figure 3A).

In a reciprocal manner however, localization of all the examined piRNA pathway components was unaffected in *kots* mutant gonads (Figure 3B). These results suggest that *kots* is a downstream component in the assembly of ping-pong piRNA pathway components at the nuage.

We next investigated the physical interaction between Kots and the piRNA components. Myc-tagged Kots expressed in germline cells was immunoprecipitated using the anti-Myc antibody in ovarian lysates (Figure 3C). While PIWI proteins involved in the ping-pong cycle, Aub, and Ago3 were found in the precipitate, Argonaute-1 (Ago1), which is involved in the miRNA but not the piRNA pathway (Yang et al., 2007), was undetectable in the Kots-enriched fraction, demonstrating selective interaction between Kots and the PIWI clade proteins. In addition, Vas, a DEAD-box helicase and crucial piRNA component (Malone et al., 2009), was also detected, further supporting that Kots interacts with components of the nuage.

### *Kots* Is Required for Proper piRNA Biogenesis

**FIGURE 4:**
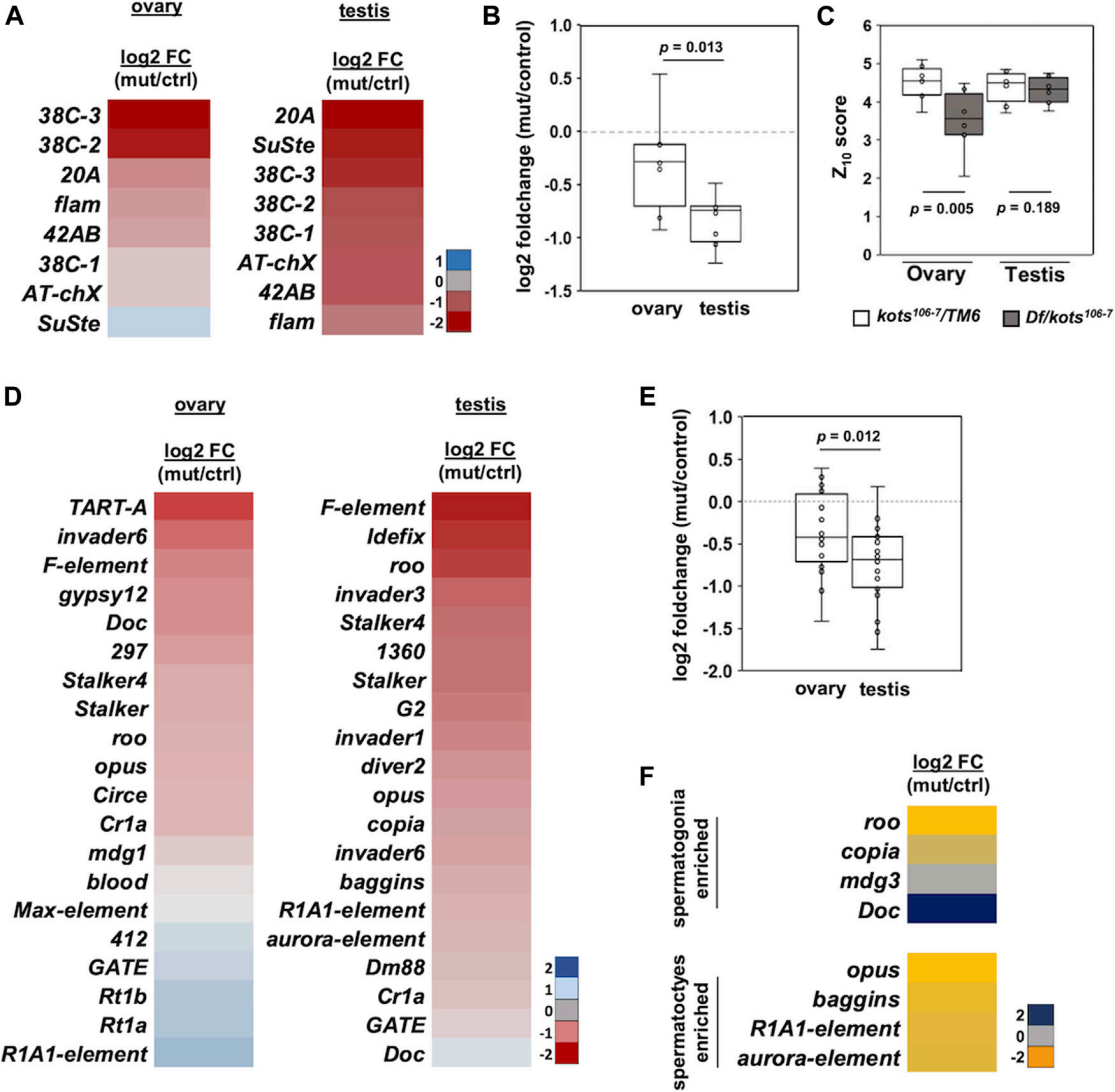
piRNAs mapping to canonical piRNA clusters and transposons are downregulated in *kots* mutants. **(A)** Log_2_ fold change (mutant/control) of piRNAs mapping to 8 major clusters in mutant and control gonadal samples. **(B)** Downregulation of cluster-mapping piRNAs is more severe in testes than that in ovaries (*t*-test, *p*-value 0.013). **(C)** Boxplot showing Z_10_ score of piRNAs mapping to clusters in ovaries and testes (*t*-test, *p*-values 0.005 and 0.189, respectively). **(D)** Log_2_ fold change of top 20 most abundant TE-mapping piRNAs. **(E)** Comparison of the top 20 most abundant TE-mapping piRNAs in ovaries and testes (*t*-test, *p*-value 0.012). **(F)** Log_2_ fold change in abundance of piRNAs mapped to TEs enriched in spermatogonia and spermatocytes.

To investigate the defects in the piRNA pathway resulting from the loss of *kots*, small RNAs from ovaries and testes were extracted and analyzed using next-generation sequencing (Supplementary Figure 3). Reads of 23–29 nucleotides in length corresponding to the piRNA population were mapped to major piRNA clusters such as the dual-stranded clusters (*42AB* and *38C*), uni-stranded clusters (*flam* and *20A*), and repeat sequences (*AT-chX* and *Su(Ste)*) (Supplementary Table 2). In both ovaries and testes of *kots* mutant, piRNAs mapping to all three groups showed a moderate reduction in abundance (Figure 4A, Supplementary Figure 4A). *kots* mutant testes exhibited a more severe reduction in the relative piRNA abundance, with a mean log_2_ fold change of −0.83 compared to −0.30 in ovaries (*t*-test, *p*-value = 0.013) (Figure 4B). Despite the reduction of piRNAs in *kots* mutant testes, the Z_10_ score, a measure of the degree of overlap between Ago3- and Aub-bound piRNAs and hence an indicator of the ping-pong cycle, was not significantly affected in the mutant testes compared to the ovaries (Figure 4C). These results suggest that there could be an alternative mechanism of piRNA biogenesis in the testes, which may involve *kots*. By mapping the piRNAs to each cluster locus, we observed a near-complete abrogation of sense and antisense piRNAs mapping to the *42AB* and *20A* clusters in mutant testes, as compared to the ovaries (Supplementary Figure 4B). In addition, piRNAs derived from the testis-specific *Su(Ste)* loci, the most abundant piRNAs comprising 28.3 and 23.4% in control and mutant testes, respectively, were reduced by half in the mutant, explaining the derepression of *ste* mRNA in the *kots* mutant (Supplementary Figures 4A, C). To investigate whether the loss of *kots* affects the production of *Su(Ste)* piRNA precursors, we verified its expression using quantitative PCR (Supplementary Figure 4D). The steady-state level of *Su(Ste)* precursor transcripts in *kots* mutant testes was comparable to that in the control, suggesting that *kots* is not required for the production of *Su(Ste)* precursor transcripts.

Next, we analyzed the piRNAs mapping to canonical transposons. Similar to the cluster-mapping piRNAs, the loss of *kots* resulted in an overall moderate but significant reduction in piRNAs against TEs in both gonads, with more severe defects in the testes (Figures 4D, E, Supplementary Figures 5A, B, Supplementary Table 2). Comparison of the top 20 most abundant TE-mapping piRNAs showed a mean −0.76 and −0.36 log_2_ fold change in testes and ovaries, respectively (*p*-value 0.012) (Figure 4E). To further understand how *kots* affects TE regulation in the testis, we selected and analyzed piRNAs mapping to transposons which were reported to be expressed in the spermatogonia and spermatocytes, as described in a previous study (Quénerch’du et al., 2016). Except for *Doc*, piRNAs in both cell populations were downregulated in mutant testes, suggesting that *kots* is involved in piRNA production in these two stages of spermatogenesis (Figure 4F). We further investigated the effects on piRNA cluster transcripts in the absence of *kots*. Similar to the case for the *Su(Ste)* piRNA precursor, the steady-state level of piRNA cluster transcripts were unaffected in *kots* mutant testes (Supplementary Figure 4D), implying that *kots* is dispensable for transcription of piRNA precursors but functions for downstream processing and/or maturation of piRNAs.

As piRNAs guide PIWI proteins to cleave TE transcripts, thereby silencing their activity in the germline, perturbations in piRNA production could be expected to alter the abundance of TEs. We conducted mRNA-seq to analyze the ovarian and testicular transcriptomic profiles and found 116 and 411 differentially expressed genes (Wald test, *p*-adj < 0.05) in *kots* mutant ovaries and testes, respectively (Supplementary Table 3). Mapping of reads to the *kots* locus confirmed the absence of functional mRNA in both gonads (Supplementary Figure 6). Following verification of the loss-of-function allele, we analyzed the expression of piRNA pathway components in *kots* mutant gonads. The expression of the most robust piRNA components was not significantly affected by the loss of *kots* in the ovaries (Wald test, *p*-adj > 0.05), indicating that *kots* does not predominantly regulate the expression of piRNA-related genes (Supplementary Figure 7).

**FIGURE 5:**
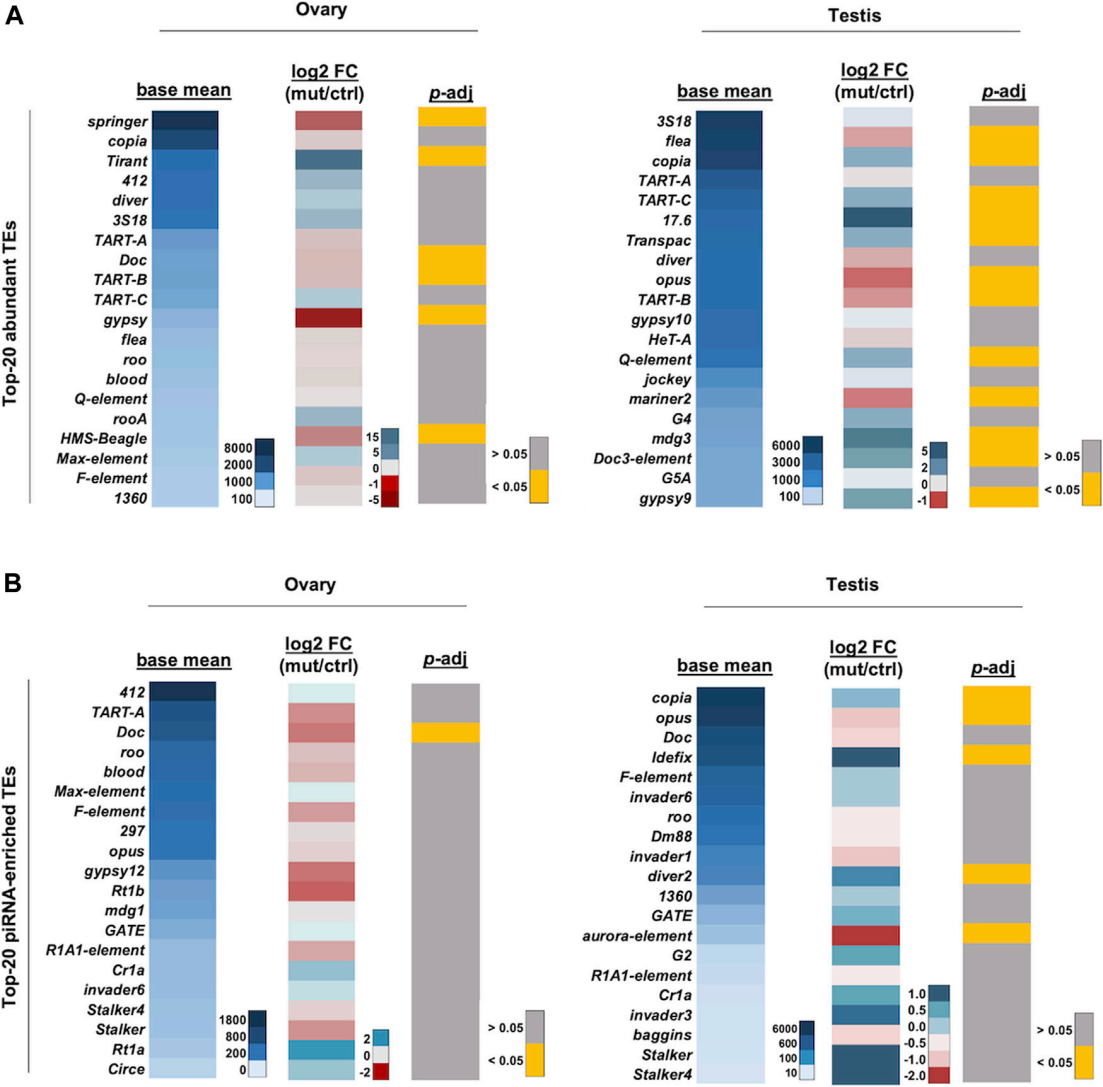
TE expression profile in *kots* mutant gonads. **(A)** Heatmaps showing the averaged read counts between the control and mutant (left panel), log_2_ fold change (mutant/control; middle panel), and *p*-adjusted values (right panel) of the top 20 most abundant TE mRNAs in ovaries and testes, respectively. **(B)** Heatmaps as in **(A)** showing corresponding values for the top-20 piRNA-enriched TEs.

The expression of TE transcripts was also analyzed. As previously reported by Chen et al. (2021), *copia*, *3S18/BEL*, and *diver* were highly expressed in the gonads and were among the most abundant TEs in our analysis (Figure 5A). Among the top 20 abundant TEs, expression of six and 12 TEs were significantly affected in ovaries and testes, respectively (*t*-test, *p*-adj < 0.05) (Figure 5A). Among the 12 TEs which were significantly affected in testes, eight were upregulated. Similarly, TEs corresponding to the top 20 most enriched piRNAs were also analyzed, and we found that TE expression was more significantly affected in the testes than in the ovaries (Figure 5B). Among the five TEs which were significantly affected in testes, three were upregulated, suggesting that *kots* is involved in the suppression of some TEs in the male germline, albeit to a milder extent than other robust components in the piRNA pathway (Vagin et al., 2004; Lim and Kai, 2007; Li et al., 2009a; Patil and Kai, 2010). Nevertheless, as with mutants of other piRNA pathway components, *kots* mutant did not exhibit a strong correlation between the reduction in piRNAs and upregulation of corresponding TEs (Nagao et al., 2010; Anand and Kai, 2012). Furthermore, it has been shown that the endo-siRNA pathway also functions to repress TEs in fly ovaries, heads, and somatic cell lines, providing another layer of the defense against TEs (Chung et al., 2008; Czech et al., 2008; Ghildiyal et al., 2008; van den Beek et al., 2018). Such functions of siRNAs and/or other mechanisms of TE repression might contribute to the imperfect correlation of piRNA reduction and TE upregulation in *kots* mutants.

We conducted gene ontology (GO) analysis to further understand the role of *kots* in the cellular process. The list of differentially expressed genes (DEGs) in *kots* mutant gonads were applied to the overrepresentation test by PANTHER and examined for enrichment in biological processes (Thomas et al., 2003). Among 411 DEGs in *kots* mutant testes (*t*-test, *p*-adj < 0.05), 188 had uniquely assigned IDs in the GO and were significantly enriched in “response to stimulus” and “multicellular developmental process” (FDR <0.05) (Supplementary Table 4). In *kots* mutant ovaries, by contrast, 74 uniquely mapped IDs among 116 DEGs (*t*-test, *p*-adj < 0.05) showed enrichment in “developmental process” and “multicellular organismal process” (FDR <0.05). We noted that some genes, including the long non-coding RNAs (lncRNAs), did not have any mapped IDs and were excluded from the GO analysis. Overall, 63 lncRNA genes, accounting for 15.3% of the 411 DEGs, were identified in the testes (*t*-test, *p*-adj < 0.05). For example, *CR43263* showed a mean 10.3 log_2_ fold change upregulation, while *CR45389* was downregulated by -7.1 log_2_ fold change (Supplementary Figure 8). In our mRNA-seq analysis, 1,279 lncRNAs were detected in the testis libraries, accounting for 8.7% of the total genes detected (*N* = 14, 698). The 1.7-fold enrichment in *kots* testicular DEGs (15.3%, compared to 8.7%) suggests that *kots* may regulate some of the lncRNAs.

## Discussion

In this study, we demonstrated the role of the *Drosophila* Tdrd1 ortholog, Kots, in piRNA biogenesis and TE regulation where the absence of *kots* resulted in male-dominant defects (Figures 2A, B, Figures 3A, B). The moderate, yet significant, defects in piRNA biogenesis suggest that *Drosophila* Tdrd1 is functionally distinct from its vertebrate counterparts, which were demonstrated to be crucial for piRNA biogenesis and fertility (Chuma et al., 2006; Huang et al., 2011). This raises the need to understand the possible coordination of Kots with the other *Drosophila* Tdrd1 orthologs, such as Vreteno, in their roles in the piRNA pathway.

We also showed the spatiotemporal localization of Kots in the male germline and demonstrated the dynamic localization of Kots from the perinuclear nuage to piNG-bodies as germline cells differentiate from spermatogonia to spermatocytes, respectively (Supplementary Figure 1).

In *Drosophila* testes, it is known that the expression of Ago3 is mostly restricted to the spermatogonia, while Aub can be found until the spermatocytes stage (Quénerch’du et al., 2016). Based on this and other studies, it has been proposed that while the heterotypic ping-pong cycle takes place between Ago3 and Aub in spermatogonia, spermatocytes may largely rely on homotypic ping-pong with Aub alone (Zhang et al., 2011). Hence, the disappearance of Kots from the perinuclear nuage and appearance of cytoplasmic condensates, piNG-bodies, during the transition from spermatogonia to spermatocytes could be relevant to alternative ping-pong mechanisms in the male germline (Kibanov et al., 2011).

piNG-bodies are large nuage-associated granules in the spermatocytes, with Ago3 at the core of the structure surrounded by Vasa and Aub (Kibanov et al., 2011). In our study, we showed that Kots is juxtaposed with Ago3 in the testes (Supplementary Figure 1). Consistently, Ago3, Aub, and Vasa were found among the interactors of Kots, providing further evidence for Kots as a component of the nuage and piNG-bodies in the spermatocytes. However, we note that the co-immunoprecipitation studies were obtained from ovarian samples and might not fully represent the molecular events in the testis. Nevertheless, given the localization of Kots in both ovaries and testes, the data from the ovarian samples should serve as a reasonable reference for further analyses using testicular samples.

While it has been shown in several studies that the loss of crucial piRNA pathway components such as *tej* (Patil and Kai, 2010) and *aub* (Nagao et al., 2010) could lead to the disorganization of the nuage and defects in piRNA biogenesis including the derepression of *ste*, the loss of *kots* does not lead to severe disorganization of the nuage in our study. Nevertheless, the upregulation of *ste* in *kots* mutant and the mislocalization of Kots in the absence of other piRNA components suggest its functional role, albeit less essential, in piRNA biogenesis. We speculate that Kots could play a supporting role to enhance the efficiency of piRNA biogenesis, either with other nuage components or those of piNG-bodies. Although the biological function of piNG-bodies remains elusive, the molecular evidence of Kots condensate in the spermatocytes could shed light on the structure and function of this unique cytoplasmic organelle in the male germline.

It has been previously reported that protein factors involved in ping-pong amplification exhibit hierarchical relationships in their localization to the nuage. Vasa, an RNA helicase that is involved in early germ cell specification and oocyte development, is required for the assembly of other components from the nuage (Findley et al., 2003; Lim and Kai, 2007). In addition, the loss of key PIWI family member, *aub*, causes the formation of large cytoplasmic aggregates comprising Krimp and dispersion of Ago3, both of which are involved in executing the heterotypic ping-pong amplification (Nagao et al., 2011; Sato et al., 2015). These observations imply that the epistasis of the factors involved in nuage formation may reflect their biological functions to drive the feed-forward ping-pong amplification cycle of piRNAs at this organelle. In this study, mutants of ping-pong cycle components resulted in the dispersion of Kots from the nuage in ovaries and testicular spermatogonia. In contrast, reciprocal experiments revealed no effect on the nuage localization of these proteins in the absence of Kots, implying that Kots may function downstream of these factors for piRNA biogenesis in germline cells. Considering the mild and negligible defects in piRNA biogenesis and ping-pong signature, Kots may be involved in enhancing piRNA biogenesis, while it is dispensable as a robust component of the ping-pong pathway.

Interestingly, *kots* mutant testes not only exhibited a reduction in piRNAs but also showed changes in the expression of other RNAs, particularly the lncRNAs (Supplementary Figure 8). Some, but not all, lncRNAs have been reported to serve as precursors of piRNAs or are expressed in tissues where piRNA pathway activities are low (Ha et al., 2014). Our observation of the enrichment of several lncRNA genes in *kots* mutant testes brings up the possibility that *kots* may regulate some lncRNAs. However, no small RNA reads were mapped to the enriched lncRNAs in *kots* mutant testes (Supplementary Table 5), raising the question of potential crosstalk between regulation of lncRNAs and piRNA biogenesis *via kots*. The relevance of these defects resulting from the loss of *kots* awaits future studies.

In summary, our study sheds light on the unique formation of Kots condensates and its male-dominant role in piRNA biogenesis in the *Drosophila* germline, providing a basis for understanding the relevance of spatiotemporal regulation of Tudor domain-containing proteins in piRNA biogenesis in future studies.

## Materials and Methods

### Fly Stocks

All stocks and crosses were raised at 25°C on standard food. The fly stocks used in this study were as follows: *ago3* ^*t2*^ (BDSC 28296), *ago3* ^*t3*^ (BDSC 28279), *aub* ^*HN2*^ (BDSC 8517), *aub* ^*QC42*^ (BDSC 4986), *piwi* ^*1*^ (BDSC 43637), *piwi* ^*2*^ (BDSC 43319), *Df(2L)BSC299* (BDSC 23683), *Df(3R)Exel8162* (BDSC 7981), *krimp* ^*f06583*^ (BDSC 18990), *Df(3R)Exel6171* (BDSC 7650), and *bam* ^*∆86*^ (BDSC 5427). Other fly stocks used were as follows: *can* ^*12*^ (Hiller et al., 2001), *tej* ^*48-5*^ (Patil and Kai, 2010), *vasa* ^*PH165*^ (Styhler et al., 1998), *spnE* ^*616*^ (Tearle and Nusslein-Volhard, 1987), *NGT40* (Tracey et al., 2000), *NGT40; nos-Gal4-VP16* (Grieder et al., 2000), and *bam-Gal4* (Chen and McKearin, 2003).

### Generation of Fly Strains

*CG9925* ^*106−7*^ *(kots* ^*106-7*^ *)* loss-of-function allele was generated by imprecise excision of the P-element, *P{SUPor-P}CG9925* ^*KG01164*^ (BDSC 14883), inserted at the 5′ UTR of *CG9925*, generating a deletion of approximately 1.57-kb encompassing the 5’ UTR through part of the second exon. Deficiency line *Df(3R)6171* (BDSC 7650) was used for the cross with *kots* ^*106-7*^ to obtain transheterozygous flies for immunostaining, quantitative RT-PCR, and next-generation sequencing analyses. *y w* or *kots* ^*106-7*^ */TM6* was used as the control, as indicated.

To generate UASp*-kots-Myc*, the coding region of full-length *kots* was amplified from the EST clone RE70955 (*Drosophila* Genomics Resource Center, NIH Grant 2P40OD010949) as the template, with the primers dTdrd1-1-Fw (5′-*CAC​C-ATG​GAA​AAG​TCG​GAG​GAA-3′*) and dTdrd1-2-Rv (5′-*TGC​AAC​TGG​TGT​GTT​TAG*-3′), and was cloned into pENTR™/D-TOPO and then recombined into the *Drosophila* Gateway Vector, pPWM (Invitrogen, Carlsbad, CA) to obtain a construct expressing C-terminal tagged Kots-6xMyc. The Kots-6xMyc fragment was then amplified and introduced into the *Xba*I site in the UASp-K10-attB vector (Koch et al., 2009). The UASp-*kots-6xMyc* construct was then injected into flies carrying the attP40 site and *phiC31* integrase (BDSC 25709). The expression of Kots-6x-Myc in the germline was driven by the *NGT40*, *NGT40*, *nos-Gal4-VP16*, or *bam-Gal4* drivers, as indicated accordingly.

### Antibody Generation

A DNA fragment corresponding to 767–891 amino acids of Kots was amplified with the primers dTdrd1-antigen-Fw (5′-*CAC​CAG​CGA​AGA​TAA​GAA​CTG​GTA​TCG​C*-3′) and dTdrd1-2 Rv (5′-*TGC​AAC​TGG​TGT​GTT​TAG*-3′), and cloned into the pENTR™/D-TOPO vector. The fragment was recombined into the pDEST17 vector (Invitrogen). 6xHis-tagged Kots antigen was expressed in bacteria and purified using Nickel Sepharose High-Performance beads (Amersham Biosciences, Piscataway, NJ, United States) following the manufacturer’s protocol and was subsequently used for immunization in rabbits.

Polyclonal anti-Ago3 antibody was raised against 1–150 amino acids of Ago3 in rats. The Ago3 antigen sequence was amplified with the forward primer (5′-*CAC​CAT​GTC​TGG​AAG​AGG​AAA*-3′) and reverse primer (5′-*TTA​CAC​TTC​GTA​ATT​AAA​AA*-3′) and cloned into pENTR™/D-TOPO before recombining into the pDEST17 vector (Invitrogen). His-tagged Ago3 was expressed in *E. coli*, and the insoluble band corresponding to His-tagged Ago3 was excised from the SDS-PAGE gel and used for immunization in rats.

GST-tagged full-length Aub (a kind gift from Dr. Paul Lasko) was expressed in *E. coli*, and the insoluble band corresponding to GST-tagged full-length Aub in the SDS-PAGE gel was excised and used for immunization in guinea pigs.

### Immunostaining

Ovaries and testes were dissected in phosphate-buffered saline (PBS) and fixed in 4% (v/v) paraformaldehyde (Electron Microscopy Sciences) for 10 min on ice. The samples were washed in PBS supplemented with 0.2% (v/v) Triton X-100 for at least 30 min with several changes of solution. Blocking was performed in PBS with 4% (w/v) bovine serum albumin (BSA) and 0.2% (v/v) Triton-X for 30 min before overnight incubation with primary antibodies in PBS with 0.4% (w/v) BSA. Unbound primary antibodies were rinsed with the same washing solution for 1 h, with several changes in washing solution. Secondary antibody incubation was conducted for 2 h at room temperature and washed for 1 h before incubation with 4′6-diamidine-2′-phenylindole (DAPI, Sigma-Aldrich, MO, United States) for 10 min in PBS. After rinsing with PBS, the samples were stored and mounted on a Fluoro-KEEPER Antifade (Nacalai Tesque, Kyoto, Japan). Images of the samples were acquired using a Zeiss LSM780 confocal microscope. Images of cytoplasmic condensates in the testes were acquired using a Zeiss LSM900 Airyscan. Images were processed using Zen (Zeiss) and Fiji (Schindelin et al., 2012) software.

The antibodies used for immunohistochemistry analysis were rabbit anti-Kots (1:200, this study), rabbit anti-Stellate (1:1000, a kind gift from Dr. William Theurkauf), mouse anti-Piwi (1:10, a kind gift from Dr. Siomi), rat anti-Ago3 (1:500, this study), guinea pig anti-Aub (1:500, this study), guinea pig anti-Vas (1:2000, Patil and Kai, 2010), rat anti-Spn-E (1:200, Patil and Kai, 2010), rabbit anti-Tej (1:500, Patil and Kai, 2010), guinea pig anti-Krimp (1:2000, Lim and Kai, 2007; Lim et al., 2009), and mouse anti-c-Myc antibody (FUJIFILM Wako, Japan).

Secondary antibodies were as follows: Alexa Fluor 488-, 555-, and 633-conjugated goat antibodies at 1:200 (Molecular Probes, Eugene, OR, United States) and CF^®^633 goat antibodies at 1:1000 (Biotium, Fremont, CA, United States).

### Quantitative RT-PCR

Total RNA was extracted from ovaries and testes using TRIzol™ (Invitrogen) following the manufacturer’s instructions. Total RNA (1 μg) was treated with DNase I at 37°C for 10 min before inactivating the enzyme at 70°C for 10 min with EDTA. cDNAs were synthesized using Superscript III reverse transcriptase (Invitrogen) following the manufacturer’s protocol. Each experiment was conducted in three biological replicates, with technical duplicates. Quantitative PCR was conducted with Fast SYBR™ Green (Invitrogen) and KAPA SYBR™ Fast (KAPA Biosystems) using StepOnePlus™ (Applied Biosystems, CA, United States). Relative expression levels were normalized to those of *Actin5c*, and fold change against heterozygous controls was compared. A list of primers is provided in Supplementary Table 1.

### Low-Molecular Weight RNA Isolation

Total RNA from ovaries and testes was extracted using the miRNeasy Mini Kit (Qiagen) according to the manufacturer’s protocol. The quality and amount of purified RNAs were measured using a NanoPhotometer P330 (Implen, Germany).

From the purified fraction, 2S ribosomal RNA (rRNA) was depleted by annealing 5 μg of the elute to complementary oligonucleotide sequences (10 pmol/μl), 5′- A*GTC​TTA​CAA​CCC​TCA​ACC​ATA​TGT​AGT​CCA​AGC​AGC​ACT -3′*, in RNase-H buffer (New England Biolabs). The DNA/RNA hybrids were removed by treatment with RNase-H (New England Biolabs) for 30 min at 37°C.

Samples depleted of 2S rRNA were loaded onto 8 M urea–polyacrylamide gel (12%) and separated in 0.5 TBE buffer. Gel areas within the range of 20–30 nt (DynaMarker®, DM253, BioDynamcs Laboratory Inc.) were excised, and RNAs were eluted in 0.3 M sodium acetate overnight and precipitated in the presence of 80% (v/v) ethanol and 0.5–1 μg/μl glycogen (Nacalai). Pellets were rinsed twice in 80% (v/v) ethanol and dissolved in RNase-free water.

### Small RNA-Seq

Small RNA libraries were sequenced using Illumina HiSeq-2500 according to the manufacturer’s protocol. Two replicates were obtained from the mutant and control gonads. Adaptors from the total reads were trimmed using fastp (Chen et al., 2018). Small RNAs were aligned to the annotated non-coding RNAs (miRNA, tRNA, snRNA, and snoRNA) using Bowtie (Langmead et al., 2009). The reference RNA sequences were obtained from Flybase (Larkin et al., 2021) and used as references for Bowtie. The counts for these RNA annotations were used to estimate the library size factor with the relative log expression (RLE) method using DESeq2 for each sample (Love et al., 2014) (Supplementary Figure 3). Normalization was performed separately for female and male libraries. Subsequently, small RNA reads were mapped to the annotated non-coding RNAs and removed. Reads of 23–29 nt in length were used for further analyses.

The piRNA reads were mapped to piRNA clusters and canonical transposons using Bowtie software (Supplementary Figure 3). The genomic loci for piRNA clusters have been reported previously (Chen et al., 2021), and references to the canonical transposons were obtained from FlyBase (Release 6.26). The consensus sequence for the multiple *Su(Ste)* and *AT-chX* repeats used were according to previously described studies (Ryazansky et al., 2016; Kotov et al., 2019). For expression analyses of piRNA clusters and transposons, read counts were normalized with size factors calculated before. The expression is shown as read count per million reads (CPM) based on the control library, and *p*-adjusted values were calculated using DESeq2, based on the Wald test. The piRNA reads were analyzed using Samtools (Li H. et al., 2009), BEDTools (Quinlan and Hall, 2010) and in-house scripts for the analyses of size distribution and 1st nucleotide preference. Graphical representations for sense and antisense piRNA distribution were plotted with pyGenomeTracks (Supplementary Figures 4B, C) (Lopez-Delisle et al., 2021), and Z-scores for the cluster- and transposon-mapped piRNAs were calculated using Signature.py (Antoniewski, 2014).

### Total RNA Isolation and mRNA-Seq

Total RNA from ovaries and testes, in two biological replicates for each control and mutant, was extracted using the RNeasy Mini Kit (Qiagen) according to the manufacturer’s protocol and enriched for poly-A containing RNAs using Oligo (dT) beads, performed by the company (Veritas Genetics). mRNA libraries were constructed using the NEBNext Ultra II RNA Library Prep Kit for Illumina (New England Biolabs). Libraries were sequenced using Nova6000. Adaptor trimming and gene expression analysis were performed as described earlier.

### Immunoprecipitation and Western Blot

Ovaries from one hundred females from *y w* control and *kots-6xMyc* transgenic line were dissected and homogenized with a pestle in homemade buffer (20 mM Tris–HCl [pH7.5], 135 mM NaCl_2_, 1.5 mM MgCl_2_, 0.2% (v/v) Triton-X, and 10% glycerol) supplemented with proteinase inhibitor (Roche, Switzerland). Subsequently 20 μl of Protein A Dynabeads^®^ (Invitrogen) was incubated with c-Myc monoclonal antibody (FUJIFILM Wako, Japan) for 30 min at room temperature before ovarian lysates were added to the pre-incubated Protein A beads and c-Myc antibody mixture and incubated overnight at 4°C. Proteins were rinsed with homemade buffer and eluted from beads in buffer containing 0.125 M Tris–HCl, 4% (w/v) SDS and 0.2 M 1,4-dithiothreitol (DTT) at 95°C. Immunoblotting was performed using the antibodies listed below, developed with Chemi-Lumi One (Nacalai Tesque, Japan), and imaged by ChemiDoc™ Touch MP (Bio-Rad). The membrane was stained with Coomassie brilliant blue for 1–2 h at room temperature after immunoblotting, for the loading control.

The primary antibodies used for Western blotting were as follows: rabbit anti-Kots (1:200; this study), rat anti-Ago3 (1:200; this study), guinea pig anti-Aub (1:1000; this study), mouse anti-Piwi (1:100; P4D2; (Saito et al., 2006)), guinea pig anti-Vasa (1:5,000; Patil and Kai, 2010), rabbit anti-Ago1 antibody (1:1000; Abcam, Cat. ID: ab5070), and mouse anti-c-Myc antibody (1:2000; FUJIFILM Wako, Japan).

HRP-conjugated secondary antibodies used for the Western blot analysis were as follows: goat anti-rat IgG (1:1000; DAKO, Glostrup, Germany), goat anti-rabbit IgG (1:3,000; Bio-Rad, Hercules, CA, United States), goat anti-guinea pig IgG (1:1000; DAKO), and goat anti-mouse IgG (1:3,000; Bio-Rad).

## Data Availability

Our RNAseq data is available from the DNA Data Bank of Japan (DDBJ). BioSample accession(s): SAMD00414018–SAMD00414033.

## Supplementary Material

Supplementary Figure 1 CG9925/Kots localizes to piNG-bodies in spermatocytes. **(A)** Immunostaining of nuage component Ago3 (green) and CG9925/Kots (red), in *kots* heterozygous ovary. Areas were enlarged (indicated by boxes in white dashed lines) to show cells in germarium (top panel, white arrows) and stage-8 egg chamber (bottom panel). Puncta of Ago3 and Kots were observed in the germarium (yellow arrowheads). Scale bar is 20 μm, and those for enlarged images are 2 μm. **(B)** Immunostaining of Kots in spermatogonia-arrested mutants (*bam* ^Δ86^) and spermatocytes-arrested mutants (*can* ^12^). Asterisks indicate testicular hub of testes. Scale bars are 50 μm, and 5 μm in insets. **(C)** In wildtype (*y* *w*) spermatogonia (SG, top panels) and spermatocytes (SC, bottom panels), Kots localizes to nuage and piNG-bodies (yellow arrowheads), respectively, together with PIWI-family proteins, Aub and Ago3. White boxes indicate areas taken at higher magnification. Scale bars are 20 μm, and those for enlarged images are 5 μm. **(D)** Coomassie brilliant blue staining of immunoblot membrane in Figure 1D, showing equal amounts of sample loaded for each condition.

Supplementary Figure 2 Gonad morphology and fecundity of *kots* mutants. **(A)** Steady-state expression of endogenous *kots* mRNA in *kots* ^*106-7*^/*TM6* and *Df*/*kots* ^*106-7*^ ovaries and testes, normalized against *Actin5c* (*n* =3, *t*-test, *p*-value < 0.005, error bars indicate s.d.). **(B)** Bright field images of *kots* ^*106-7*^/*TM6* and *Df*/*kots* ^*106-7*^ ovaries and testes showed no gross morphological defects in *kots* mutants. Scale bars are 500 μm. **(C)** Hatching rates of embryos laid by *kots* ^*106-7*^/*TM6* and *Df*/*kots* ^*106-7*^ females (*n* = 4, t-test, *p*-value > 0.05, error bars indicate s.d.) **(D)** Male fertility test. Total number of functional sperms were determined from embryo hatch counts of crosses between *Df*/*kots* ^*106-7*^ males and *y* *w* virgin females over a period of 10 days (*n* = 4, *t*-test, *p*-value > 0.05, error bars indicate s.d.). Heterozygous males were used as controls.

Supplementary Figure 3 Pipeline for analysis of small RNAs libraries. Schematic diagram showing the procedure (singular) for small RNA analysis. Total RNA from two biological replicates were trimmed for adaptor sequences. Small non-coding RNAs annotated in the genome were used for normalization and removed from subsequent analyses. Remaining reads were selected by size (23–29 nt) as piRNAs and analyzed after normalization.

Supplementary Figure 4 Cluster analysis of small RNAs. **(A)** From left, heat maps showing total reads count, length distribution (percentage of total reads against respective clusters), nucleotides frequency at the first 5' position, ping-pong Z_10_-score and antisense piRNAs (percentage of antisense reads in each cluster) mapping to major piRNA clusters in ovary and testis samples. **(B)** Strand-specific mapping of piRNAs against germline and somatic *(flam)* cluster loci in control and mutant ovaries. Sense and antisense piRNAs are shown in blue and red, respectively. Plots show mapping from one biological replicate. Reads from the second biological replicate are overlayed and indicated in grey. **(C)** As in **(B)**, for mutant testes. piRNAs mapping to the testis-dominant cluster, *AT-chX*, and testis-specific cluster, *Su(Ste)* are mapped to consensus repeats sequences. **(D)** Expression levels of cluster transcripts and piRNA precursors derived from *42AB, 38C, 20A* and *Su(Ste)* locus, detected by qRT-PCR, in testes. All samples were normalized to *Actin5c* (*n* = 3, *t*-test, *p*-values > 0.05, error bars indicate s.d.).

Supplementary Figure 5 Analysis of piRNAs mapping to canonical transposons. Total reads count, length distribution, nucleotides frequency and Z_10_-score of piRNAs mapping to canonical transposons in ovary **(A)** and testis **(B)** samples. Transposons are ranked by the abundance of reads count.

Supplementary Figure 6 Loss of *kots* transcription in mutant gonads. Schematic diagram showing the reads mapping to *kots* loci in heterozygous control (green) and mutant (red). Reads are absent in from entire *kots* locus in mutant ovaries **(A)** but detected at the remaining region in testes **(B)**, suggesting a distinct regulation of *kots* transcription.

Supplementary Figure 7 Expression levels of piRNA pathway components in *kots* mutant. Log_2_ fold change of reads mapping to piRNA pathway components loci in *kots* mutant ovaries and testes.

Supplementary Figure 8 Reads mapping to representative lncRNA loci. Reads mapping to lncRNA are upregulated for *CR43263/FBtr0306546* **(A)** and downregulated for *CR45389/FBtr0345831* **(B)** in *kots* mutant testes (red) compared to the heterozygous control (green).
