# Supplementary material for: The Tudor Domain-Containing Protein, Kotsubu (CG9925), Localizes to the Nuage and Functions in piRNA Biogenesis in D. melanogaster
Source: Front Mol Biosci. 2022 Mar 29;9:818302. doi: 10.3389/fmolb.2022.818302 (PMC9002060; doi:10.3389/fmolb.2022.818302)
Supplement: Supplementary file 3 [file Table1.DOCX]

| **Primer** | **Sequence** | **References** |
| --- | --- | --- |
| CG9925/Kots_Forward | *ATGGAAAAGTCGGAGGAAAAGAC* | This paper |
| CG9925/Kots_Reverse | *GCCTCTGCCAATCCAATTTCT* | This paper |
| Stellate Forward | *GTCAGAGCGTGGCGTGATTGC* | Patil and Kai, 2010 |
| Stellate Reverse | *GGCCCGAACATCGCTCCGTCC* | Patil and Kai, 2010 |
| pre(Su(Ste))_1_Forward | *GCTGTAAAAACTACTTCCATCC* | This paper |
| pre(Su(Ste))_1_Reverse | *TGAGGACTTGGGCGATTTAAGTG* | This paper |
| 42AB_1_Forward | *CGTCCCAGCCTACCTAGTCA* | This paper |
| 42AB_1_Reverse | *ACTTCCCGGTGAAGACTCCT* | This paper |
| 38C_3_Forward | *TGACGGTCTCTATGGGCAGGC* | This paper |
| 38C_3_Reverse | *TTCAACAGCGACTGACTGCCG* | This paper |
| 20A_Forward | *GCCTACGCAGAGGCCTAAGT* | This paper |
| 20A_Reverse | *CAGATGTGGTCCAGTTGTGC* | This paper |
| Actin5C Forward | *TGCCCATCTACGAGGGTTAT* | Lim and Kai, 2007 |
| Actin5C Reverse | *AGTACTTGCGCTCTGGCGG* | Lim and Kai, 2007 |

**Supplementary Table 1.** List of primers used for qPCR.
