# Supplementary material for: The Tudor Domain-Containing Protein, Kotsubu (CG9925), Localizes to the Nuage and Functions in piRNA Biogenesis in D. melanogaster
Source: Front Mol Biosci. 2022 Mar 29;9:818302. doi: 10.3389/fmolb.2022.818302 (PMC9002060; doi:10.3389/fmolb.2022.818302)
Supplement: Supplementary file 4 [file Presentation1.pdf]

Figure S1

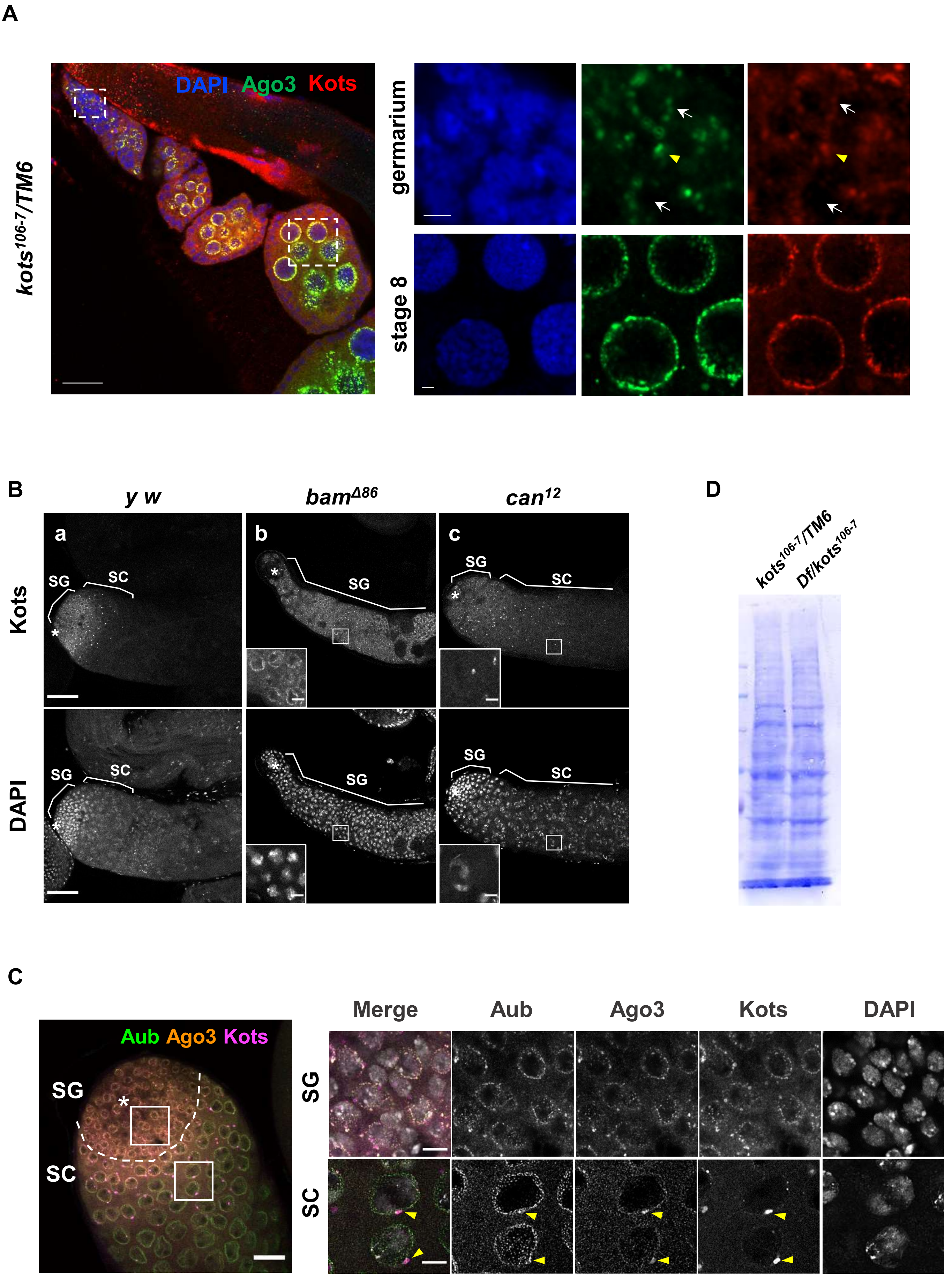
A
DAPI Ago3 Kots
kots106-7/TM6
germarium
stage 8
B
y w
bamΔ86
can12
a
b
c
SG
SC
Kots
DAPI
C
Aub Ago3 Kots
Merge
Aub
Ago3
Kots
DAPI
SG
SC
D
kots106-7/TM6
Df/kots106-7

Figure S2

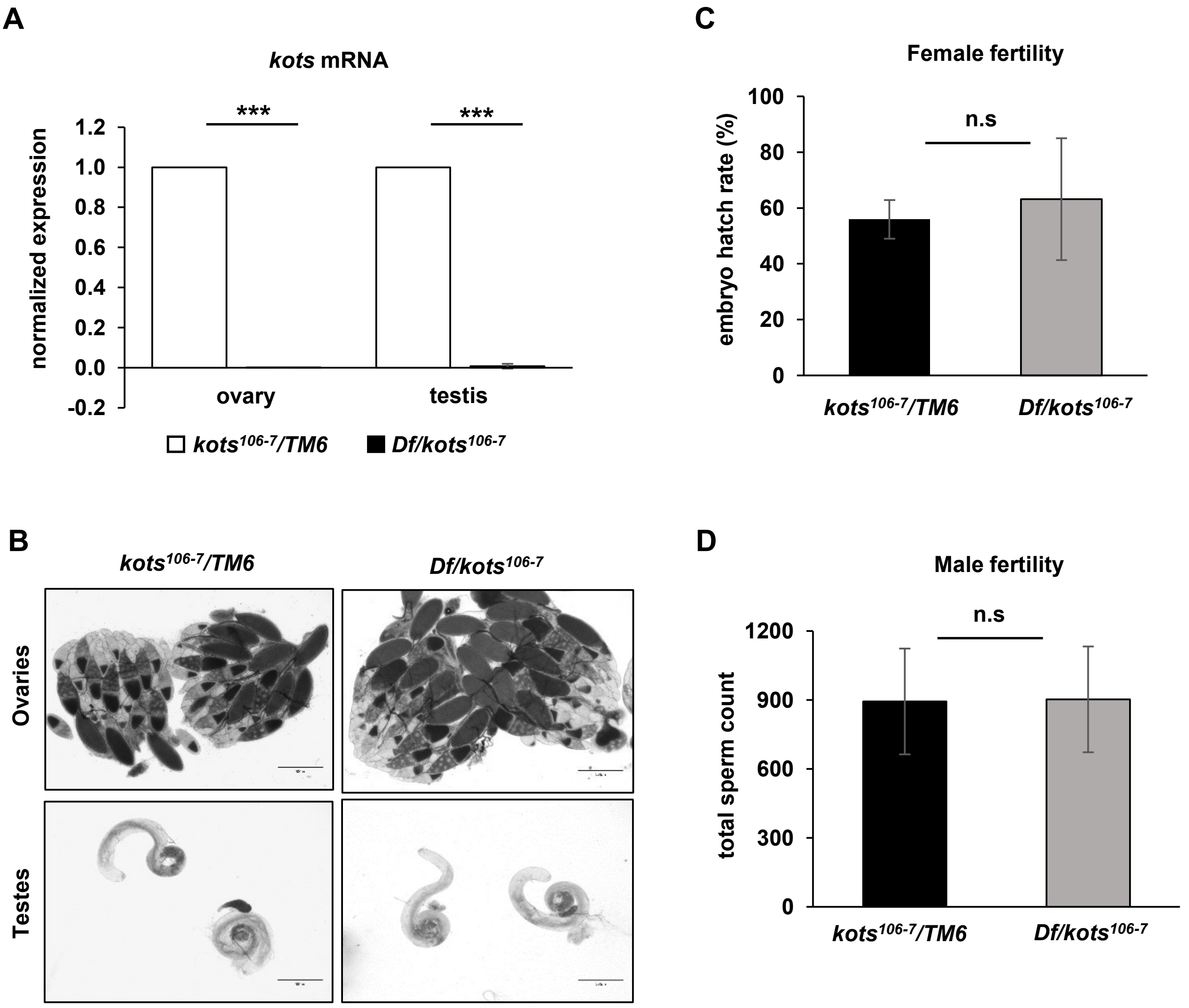

A
kots mRNA
normalized expression
***
***
1.2
1.0
0.8
0.6
0.4
0.2
0.0
-0.2
ovary
testis
kots106-7/TM6
Df/kots106-7
B
kots106-7/TM6
Df/kots106-7
Ovaries
Testes
C
Female fertility
embryo hatch rate (%)
n.s
100
80
60
40
20
0
kots106-7/TM6
Df/kots106-7
D
Male fertility
total sperm count
n.s
1200
900
600
300
0
kots106-7/TM6
Df/kots106-7

Figure S3

Pipeline for small-RNA-seq analysis

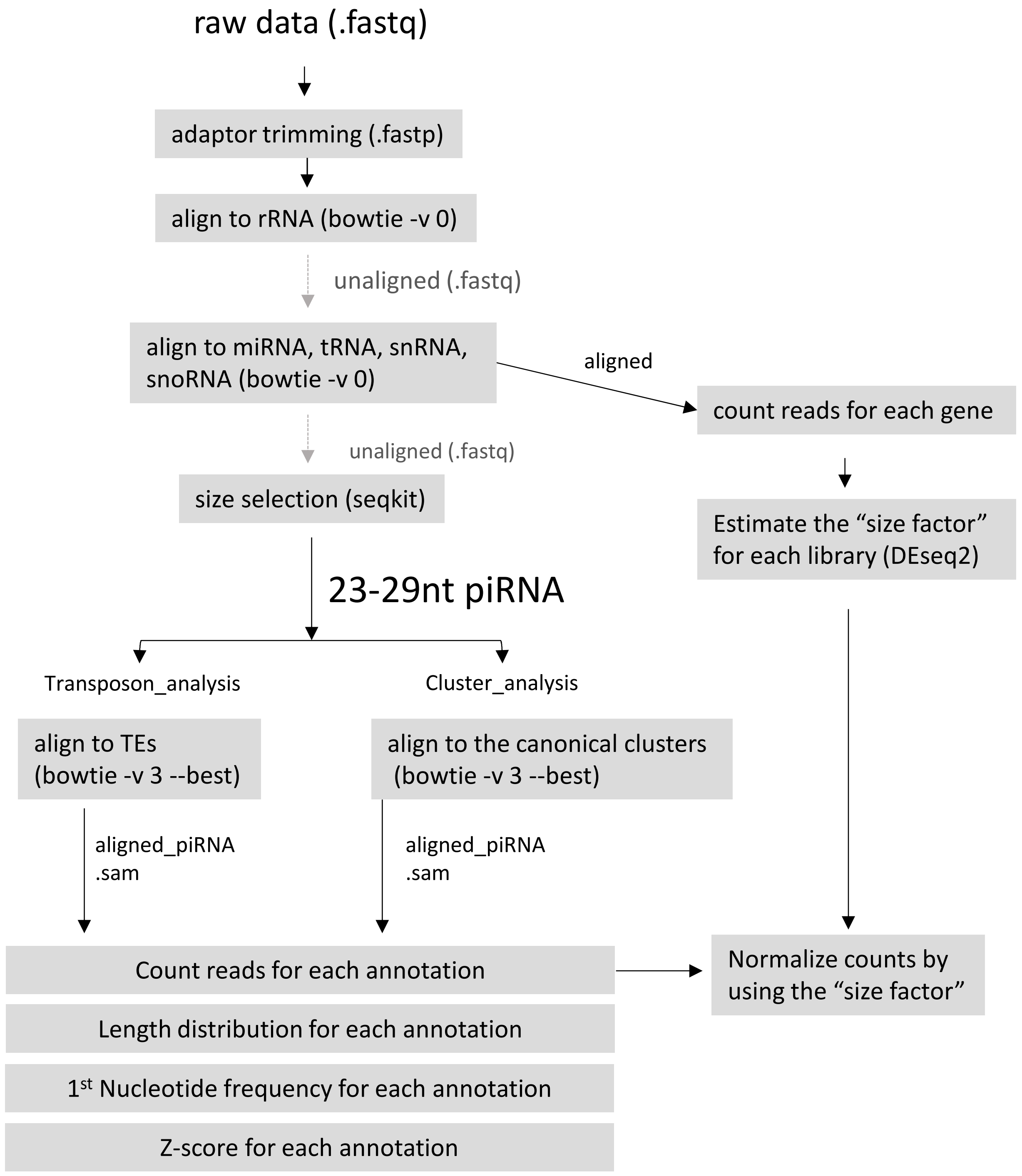

Figure S4A

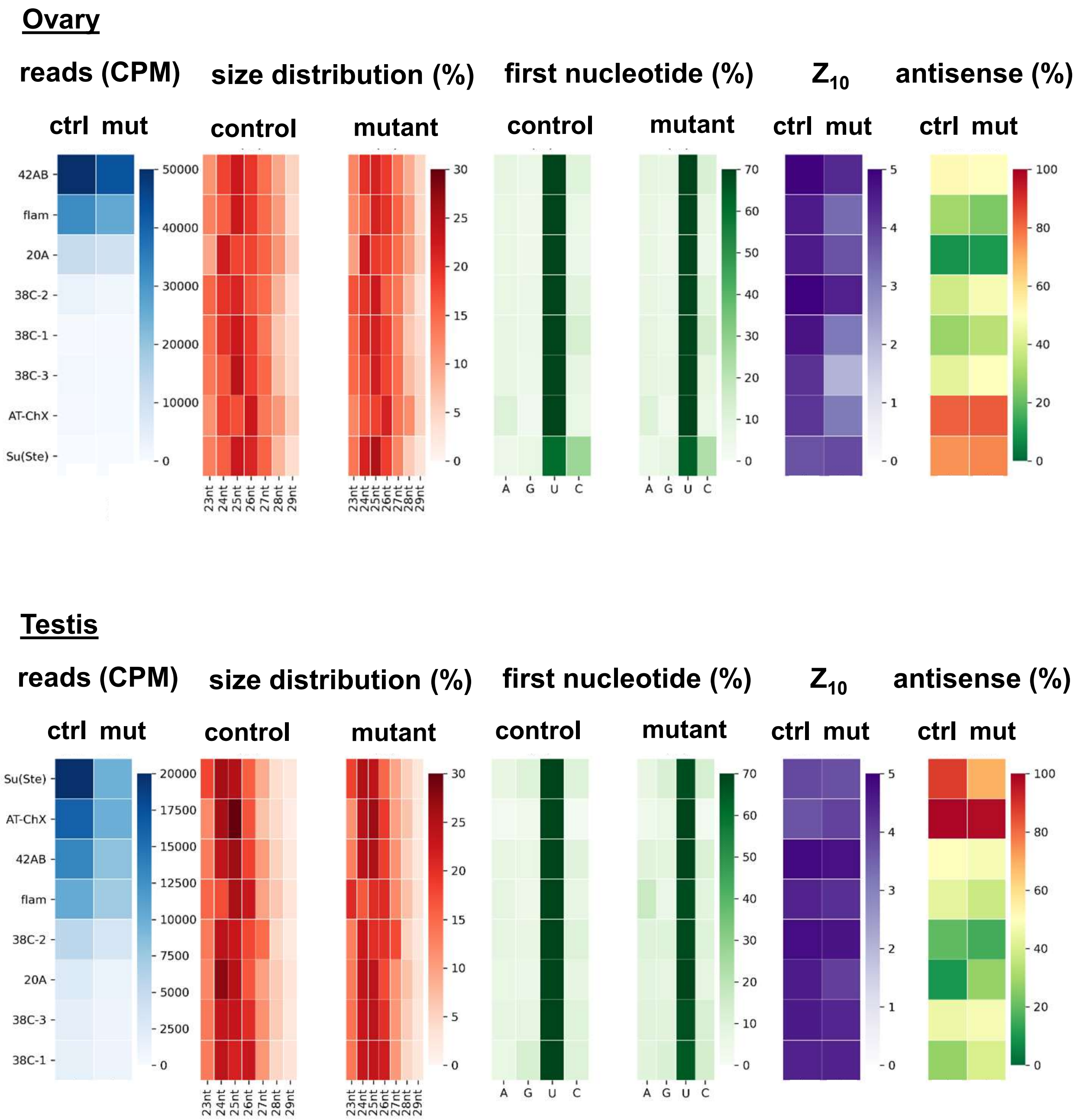

Ovary
reads (CPM)
size distribution (%)
first nucleotide (%)
Z_{10}
antisense (%)
ctrl mut
control
mutant
control
mutant
ctrl mut
ctrl mut
42AB
flam
20A
38C-2
38C-1
38C-3
AT-ChX
Su(Ste)
50000
40000
30000
20000
10000
0
23nt 24nt 25nt 26nt 27nt 28nt 29nt
30 25 20 15 10 5 0
A G U C
70 60 50 40 30 20 10 0
5 4 3 2 1 0
100 80 60 40 20 0
Testis
reads (CPM)
size distribution (%)
first nucleotide (%)
Z_{10}
antisense (%)
ctrl mut
control
mutant
control
mutant
ctrl mut
ctrl mut
Su(Ste)
AT-ChX
42AB
flam
38C-2
20A
38C-3
38C-1
20000
17500
15000
12500
10000
7500
5000
2500
0
23nt 24nt 25nt 26nt 27nt 28nt 29nt
30 25 20 15 10 5 0
A G U C
70 60 50 40 30 20 10 0
5 4 3 2 1 0
100 80 60 40 20 0

Figure S4B

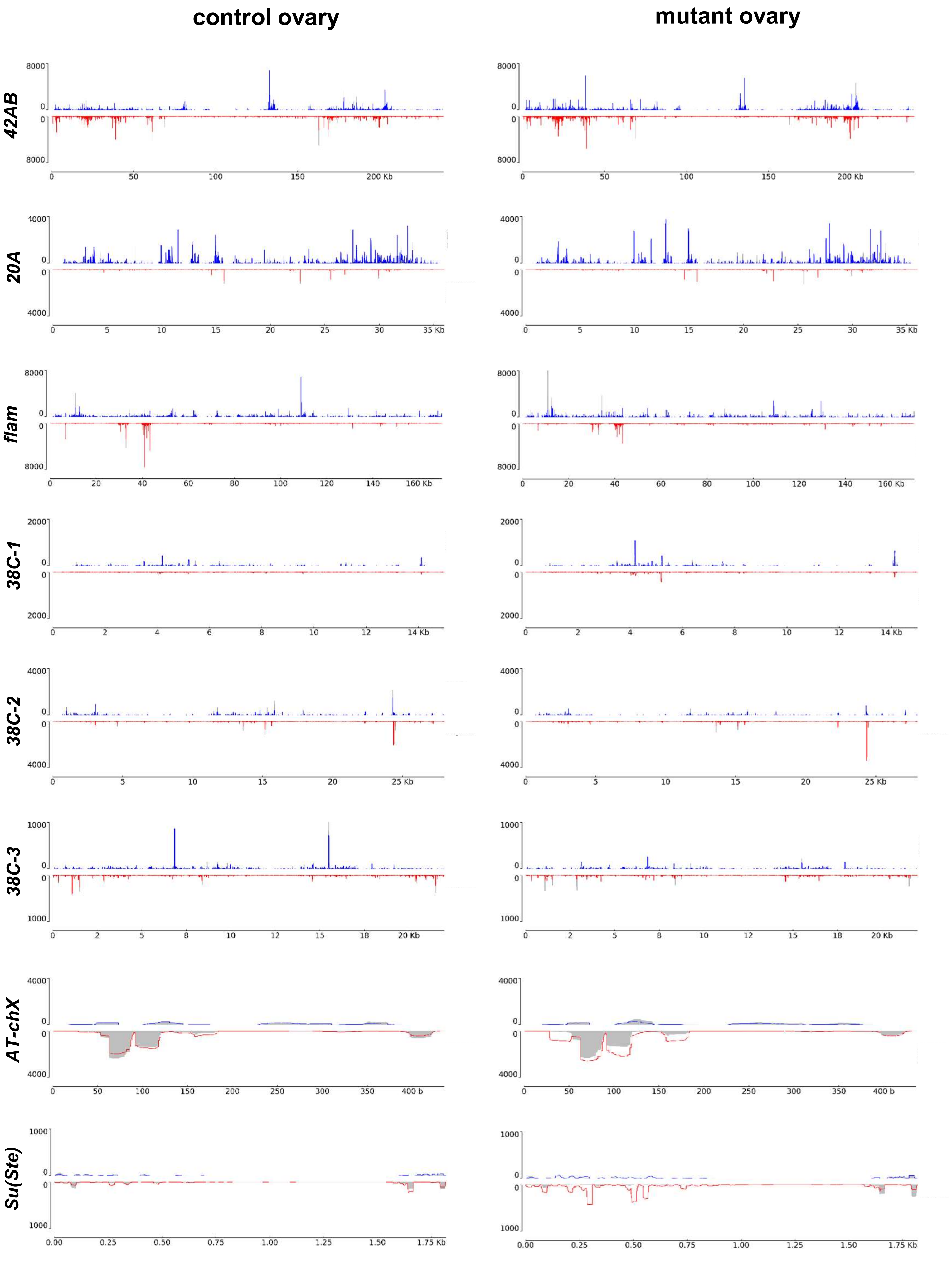
control ovary
mutant ovary
42AB
20A
flam
38C-1
38C-2
38C-3
AT-chX
Su(Ste)

Figure S4C

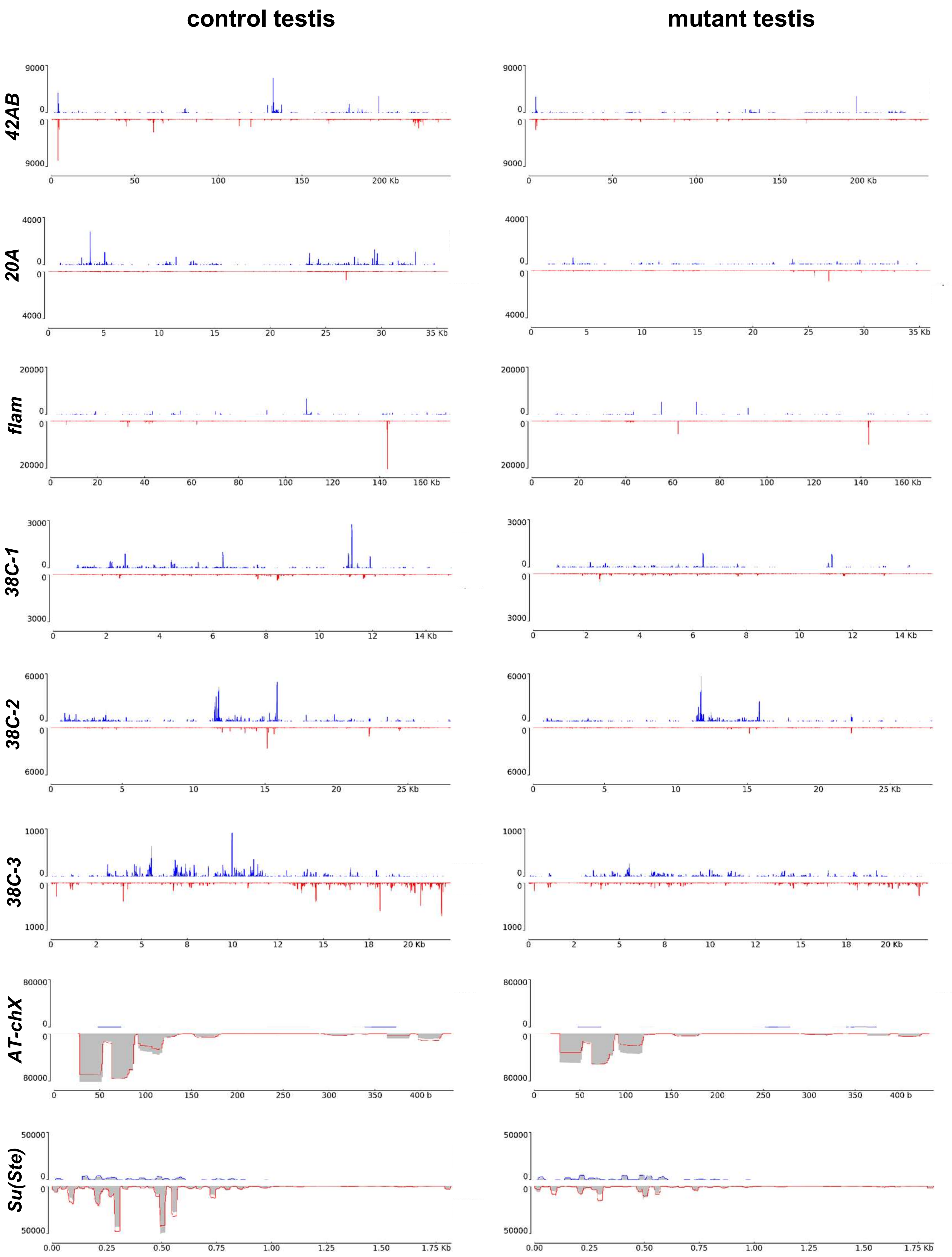
control testis
mutant testis
42AB
20A
flam
38C-1
38C-2
38C-3
AT-chX
Su(Ste)
9000
0
200 Kb
4000
35 Kb
20000
160 Kb
3000
14 Kb
6000
25 Kb
1000
20 Kb
80000
400 b
50000
1.75 Kb

Figure S4D

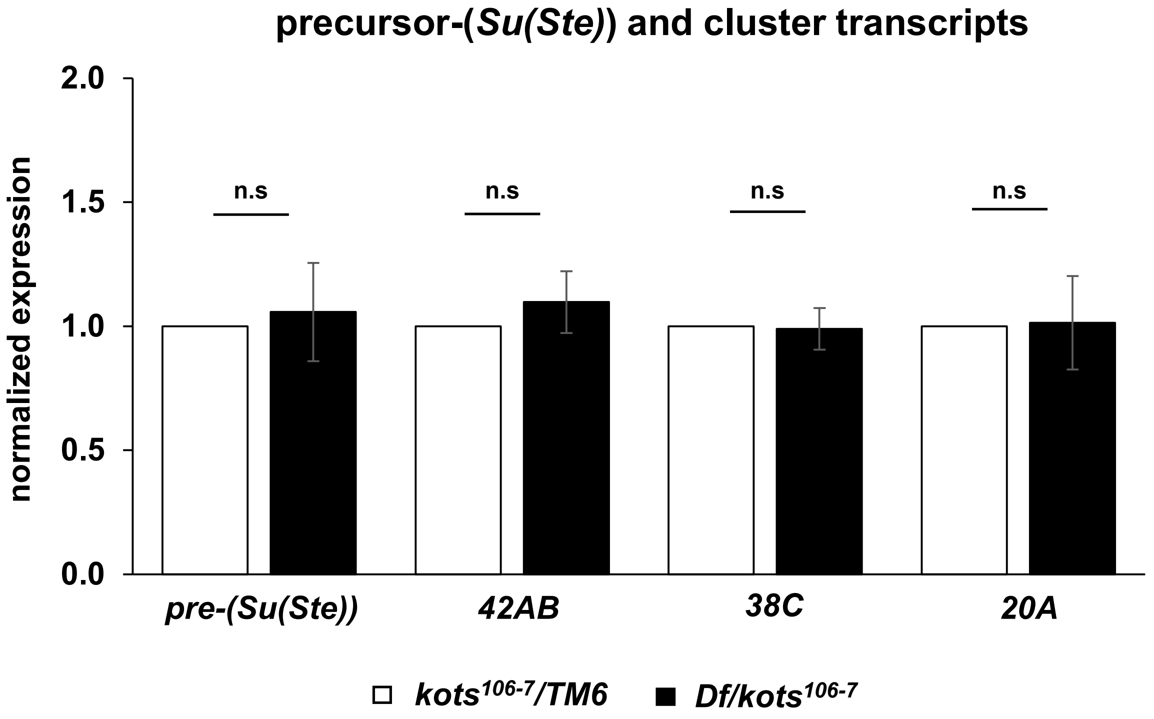
precursor-(Su(Ste)) and cluster transcripts
normalized expression
2.0
1.5
1.0
0.5
0.0
n.s
n.s
n.s
n.s
pre-(Su(Ste))
42AB
38C
20A
kots106-7/TM6
Df/kots106-7

Figure S5A

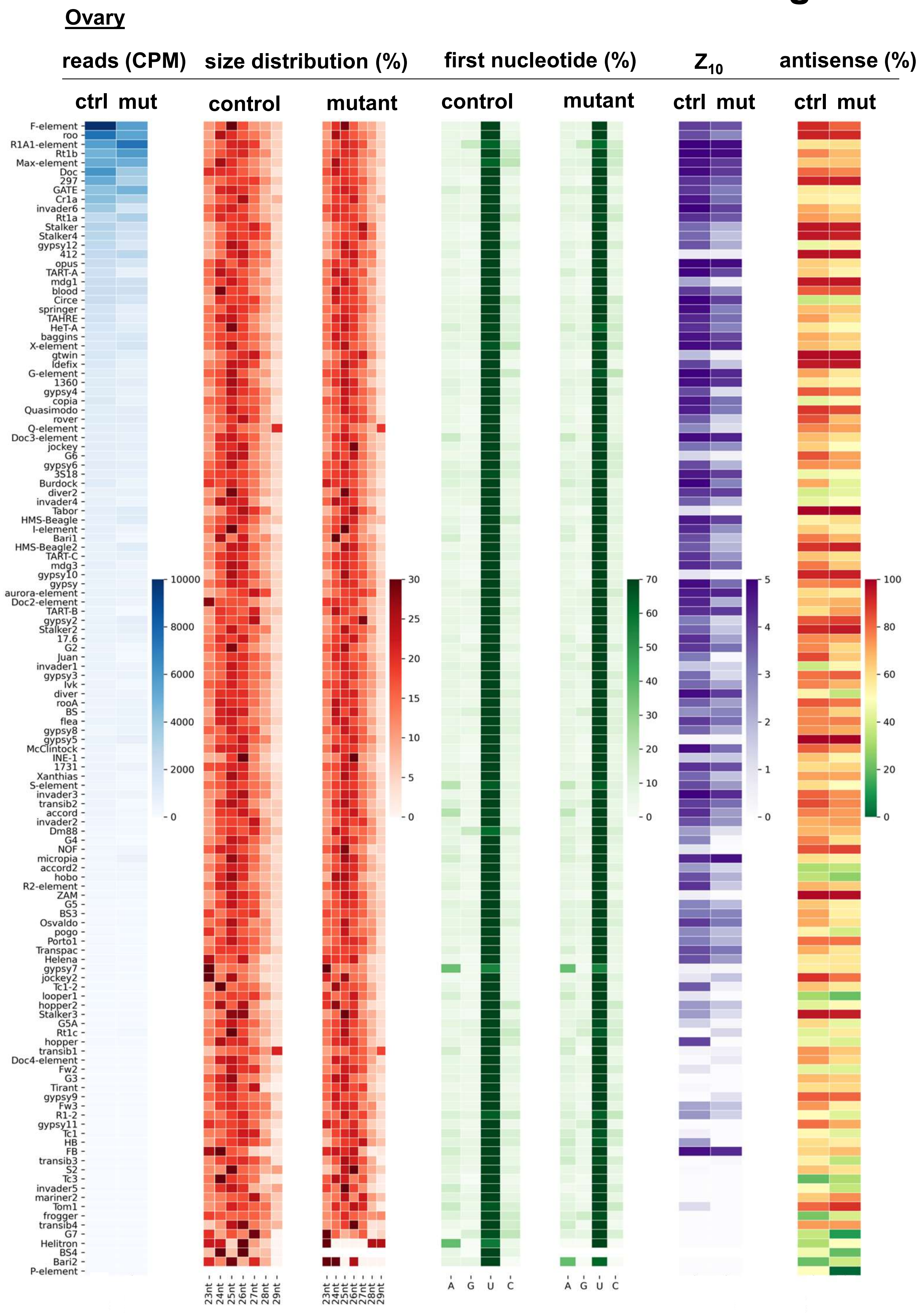
Ovary
reads (CPM)
size distribution (%)
first nucleotide (%)
Z_{10}
antisense (%)
ctrl mut
control
mutant
control
mutant
ctrl mut
ctrl mut
F-element
roo
R1A1-element
Rt1b
Max-element
Doc
297
GATE
Cr1a
invader6
Rt1a
Stalker
Stalker4
gypsy12
412
opus
TART-A
mdg1
blood
Circe
springer
TAHRE
HeT-A
baggins
X-element
gtwin
Idefix
G-element
1360
gypsy4
copia
Quasimodo
rover
Q-element
Doc3-element
jockey
G6
gypsy6
3S18
Burdock
diver2
invader4
Tabor
HMS-Beagle
I-element
Bari1
HMS-Beagle2
TART-C
mdg3
gypsy10
gypsy
aurora-element
Doc2-element
TART-B
gypsy2
Stalker2
17.6
G2
Juan
invader1
gypsy3
Ivk
diver
rooA
BS
flea
gypsy8
gypsy5
McClintock
INE-1
1731
Xanthias
S-element
invader3
transib2
accord
invader2
Dm88
G4
NOF
micropia
accord2
hobo
R2-element
ZAM
G5
BS3
Osvaldo
pogo
Porto1
Transpac
Helena
gypsy7
jockey2
Tc1-2
looper1
hopper2
Stalker3
G5A
Rt1c
hopper
transib1
Doc4-element
Fw2
G3
Tirant
gypsy9
Fw3
R1-2
gypsy11
Tc1
HB
FB
transib3
S2
Tc3
invader5
mariner2
Tom1
frogger
transib4
G7
Helitron
BS4
Bari2
P-element
10000
8000
6000
4000
2000
0
30
25
20
15
10
5
0
70
60
50
40
30
20
10
0
5
4
3
2
1
0
100
80
60
40
20
0
23nt 24nt 25nt 26nt 27nt 28nt 29nt
23nt 24nt 25nt 26nt 27nt 28nt 29nt
A G U C
A G U C

Figure S5B

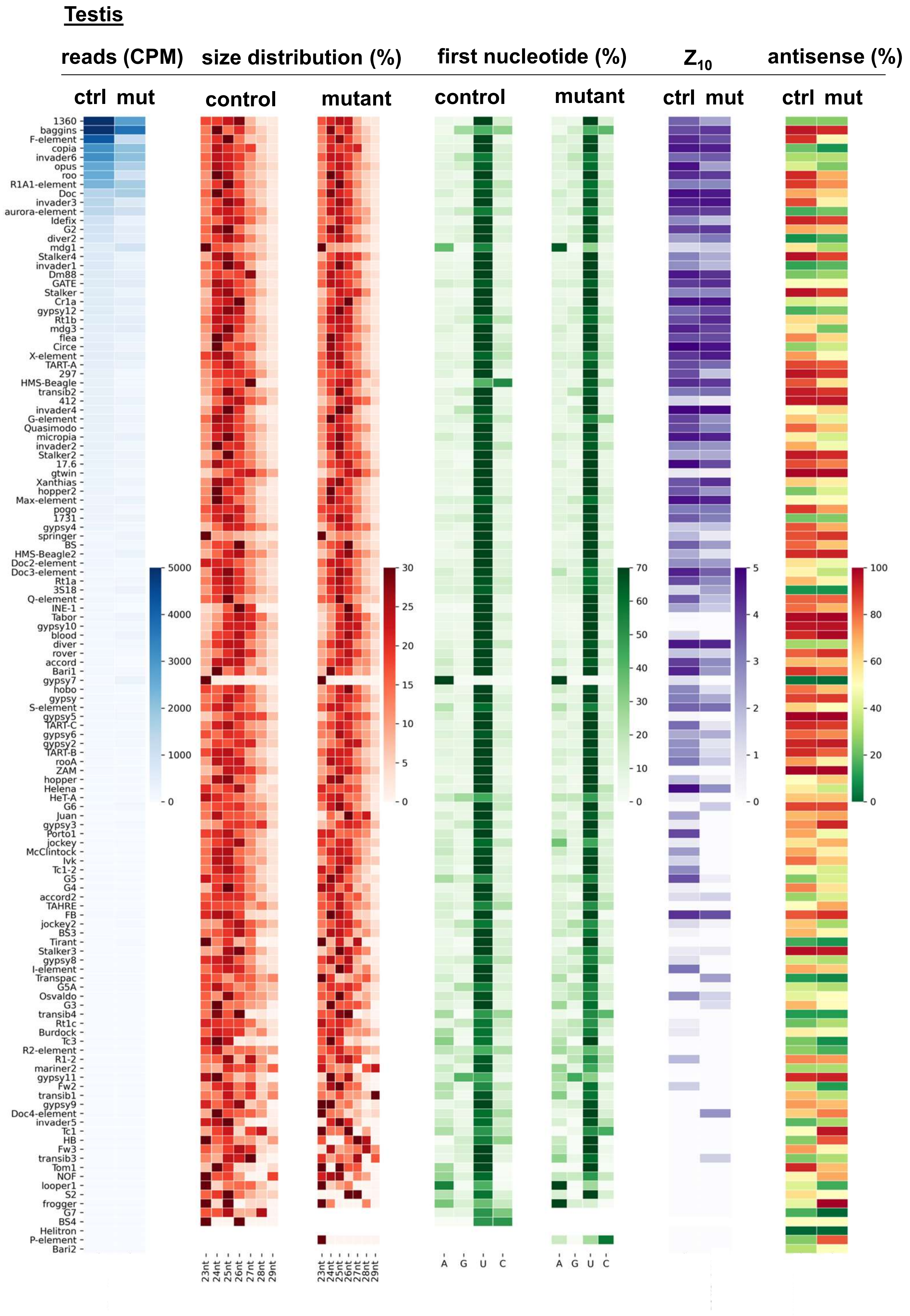

Testis
reads (CPM)
size distribution (%)
first nucleotide (%)
Z_{10}
antisense (%)
ctrl mut
control
mutant
control
mutant
ctrl mut
ctrl mut
1360
baggins
F-element
copia
invader6
opus
roo
R1A1-element
Doc
invader3
aurora-element
Idefix
G2
diver2
mdg1
Stalker4
invader1
Dm88
GATE
Stalker
Cr1a
gypsy12
Rt1b
mdg3
flea
Circe
X-element
TART-A
297
HMS-Beagle
transib2
412
invader4
G-element
Quasimodo
micropia
invader2
Stalker2
17.6
gtwin
Xanthias
hopper2
Max-element
pogo
1731
gypsy4
springer
BS
HMS-Beagle2
Doc2-element
Doc3-element
Rt1a
3S18
Q-element
INE-1
Tabor
gypsy10
blood
diver
rover
accord
Bari1
gypsy7
hobo
gypsy
S-element
gypsy5
TART-C
gypsy6
gypsy2
TART-B
rooA
ZAM
hopper
Helena
HeT-A
G6
Juan
gypsy3
Porto1
jockey
McClintock
Ivk
Tc1-2
G5
G4
accord2
TAHRE
FB
jockey2
BS3
Tirant
Stalker3
gypsy8
I-element
Transpac
G5A
Osvaldo
G3
transib4
Rt1c
Burdock
Tc3
R2-element
R1-2
mariner2
gypsy11
Fw2
transib1
gypsy9
Doc4-element
invader5
Tc1
HB
Fw3
transib3
Tom1
NOF
looper1
S2
frogger
G7
BS4
Helitron
P-element
Bari2
5000
4000
3000
2000
1000
0
30
25
20
15
10
5
0
70
60
50
40
30
20
10
0
5
4
3
2
1
0
100
80
60
40
20
0
23nt 24nt 25nt 26nt 27nt 28nt 29nt
23nt 24nt 25nt 26nt 27nt 28nt 29nt
A G U C
A G U C

Figure S6

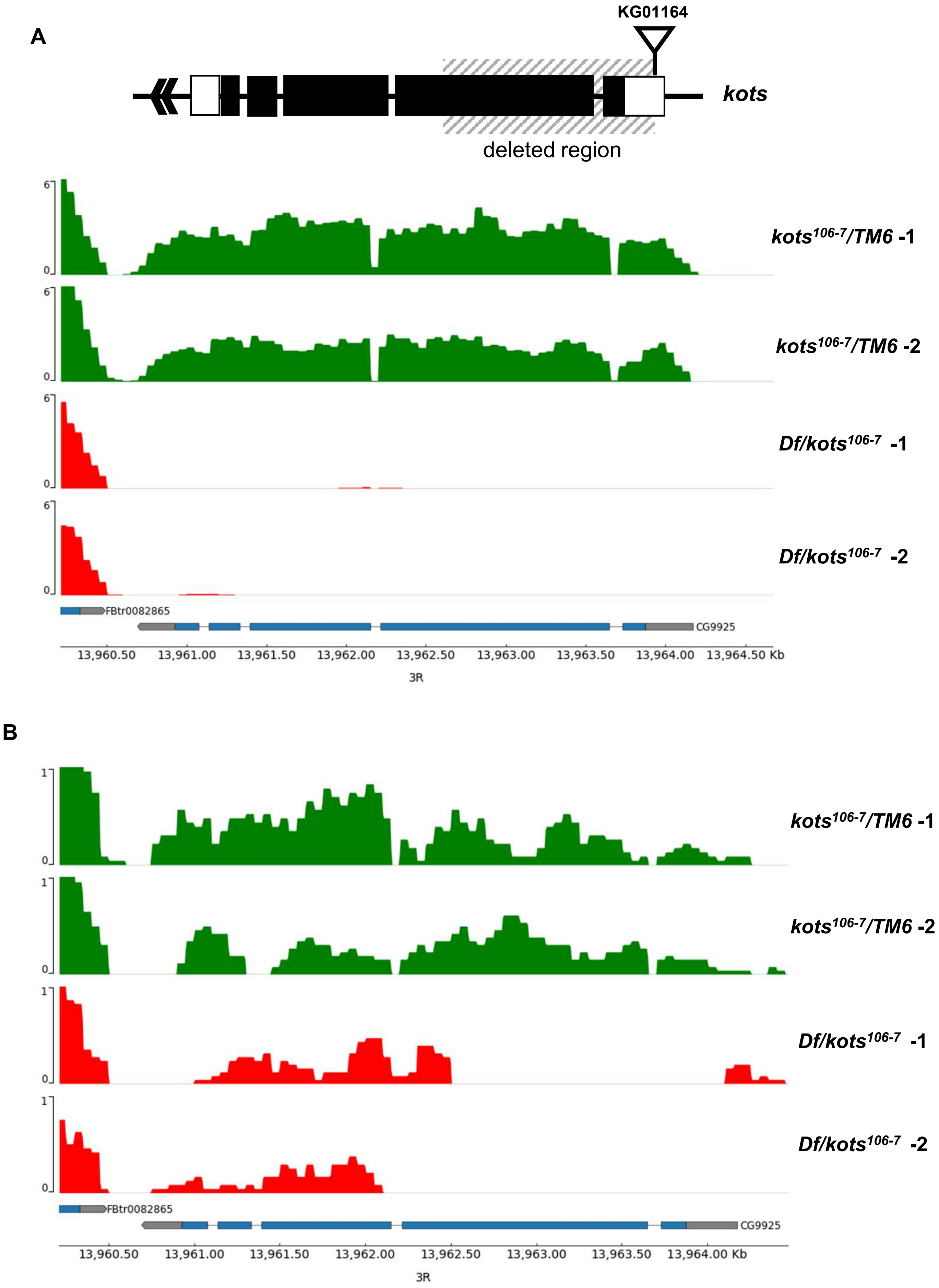

A
KG01164
kots
deleted region
kots106-7/TM6 -1
kots106-7/TM6 -2
Df/kots106-7 -1
Df/kots106-7 -2
FBtr0082865
CG9925
13,960.50
13,961.00
13,961.50
13,962.00
13,962.50
13,963.00
13,963.50
13,964.00
13,964.50 Kb
3R
B
kots106-7/TM6 -1
kots106-7/TM6 -2
Df/kots106-7 -1
Df/kots106-7 -2
FBtr0082865
CG9925
13,960.50
13,961.00
13,961.50
13,962.00
13,962.50
13,963.00
13,963.50
13,964.00 Kb
3R

Figure S7

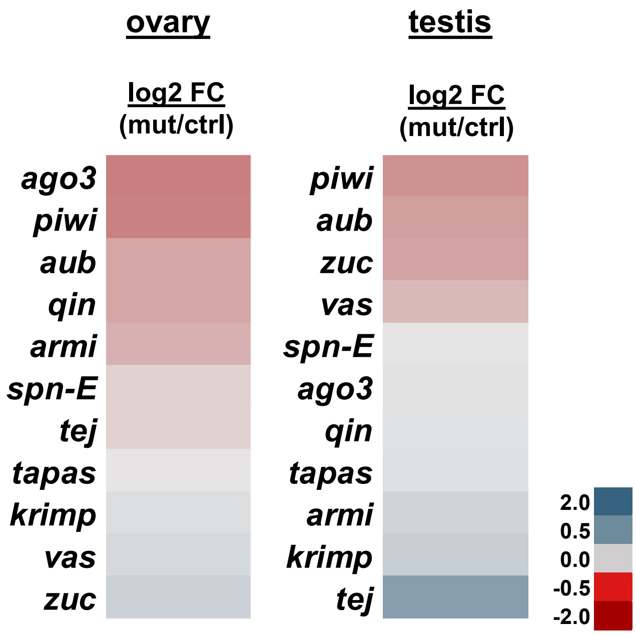

ovary
log2 FC
(mut/ctrl)
ago3
piwi
aub
qin
armi
spn-E
tej
tapas
krimp
vas
zuc
testis
log2 FC
(mut/ctrl)
piwi
aub
zuc
vas
spn-E
ago3
qin
tapas
armi
krimp
tej
2.0
0.5
0.0
-0.5
-2.0

Figure S8

A

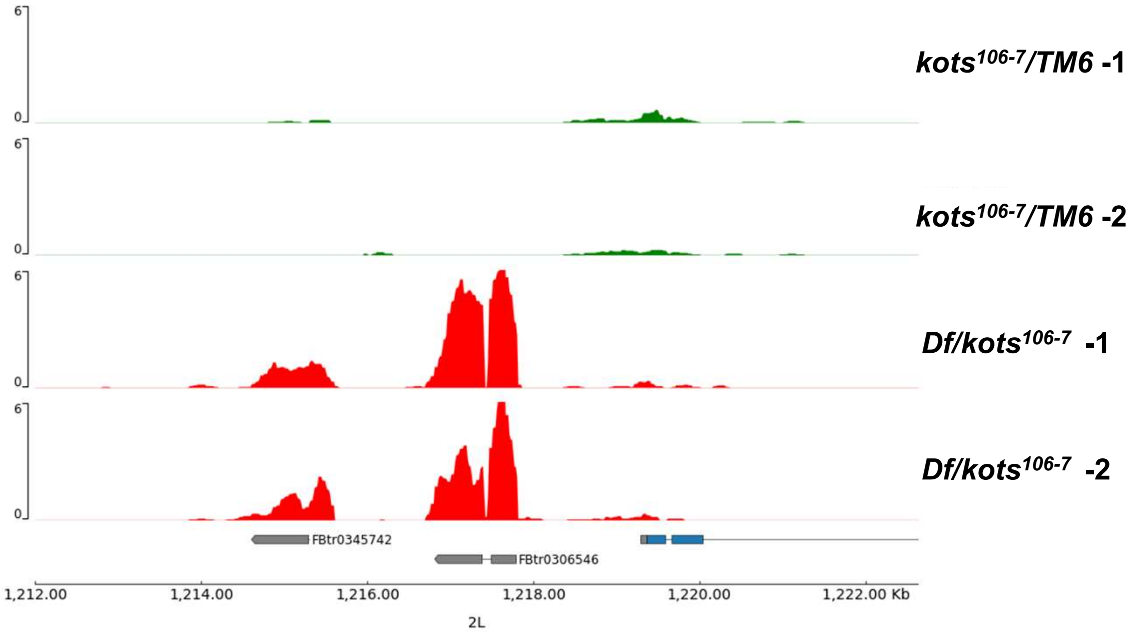

CR43263

B

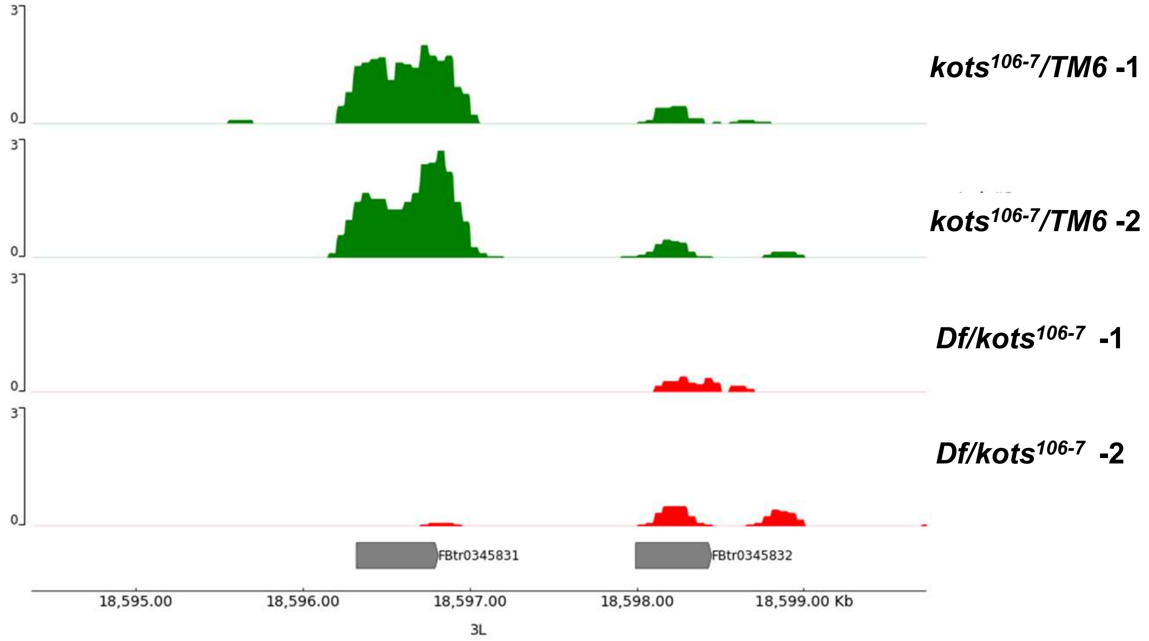

CR45389
